# Potassium Acts as a GTPase-Activating Element on Each Nucleotide-Binding Domain of the Essential *Bacillus subtilis* EngA

**DOI:** 10.1371/journal.pone.0046795

**Published:** 2012-10-08

**Authors:** Anne-Emmanuelle Foucher, Jean-Baptiste Reiser, Christine Ebel, Dominique Housset, Jean-Michel Jault

**Affiliations:** 1 Institut de Biologie Structurale, Université Joseph Fourier Grenoble 1, Grenoble, France; 2 UMR 5075 CNRS, Grenoble, France; 3 CEA, Grenoble, France; University of Lethbridge, Canada

## Abstract

EngA proteins form a unique family of bacterial GTPases with two GTP-binding domains in tandem, namely GD1 and GD2, followed by a KH (K-homology) domain. They have been shown to interact with the bacterial ribosome and to be involved in its biogenesis. Most prokaryotic EngA possess a high GTPase activity in contrast to eukaryotic GTPases that act mainly as molecular switches. Here, we have purified and characterized the GTPase activity of the *Bacillus subtilis* EngA and two shortened EngA variants that only contain GD1 or GD2-KH. Interestingly, the GTPase activity of GD1 alone is similar to that of the whole EngA, whereas GD2-KH has a 150-fold lower GTPase activity. At physiological concentration, potassium strongly stimulates the GTPase activity of each protein construct. Interestingly, it affects neither the affinities for nucleotides nor the monomeric status of EngA or the GD1 domain. Thus, potassium likely acts as a chemical GTPase-activating element as proposed for another bacterial GTPase like MnmE. However, unlike MnmE, potassium does not promote dimerization of EngA. In addition, we solved two crystal structures of full-length EngA. One of them contained for the first time a GTP-like analogue bound to GD2 while GD1 was free. Surprisingly, its overall fold was similar to a previously solved structure with GDP bound to both sites. Our data indicate that a significant structural change must occur upon K^+^ binding to GD2, and a comparison with *T. maritima* EngA and MnmE structures allowed us to propose a model explaining the chemical basis for the different GTPase activities of GD1 and GD2.

## Introduction

GTPases form one of the most widely spread protein families in all kingdoms of life and they play vital functions in many cellular processes, including translation, cellular trafficking and cell division [1], [2]. In eukaryotes, these enzymes have usually a very low intrinsic GTPase activity which can be strongly stimulated, up to several orders of magnitude, by additional protein partners known as GAP (‘GTPase Activating Protein’) [3]. Another class of GTPases has been described in which the catalytic activity is promoted upon a nucleotide-dependent dimerization [4]. In both cases, an optimal positioning of catalytic residue(s) is thereby afforded, albeit using different ways. Some residues are brought in *trans* by a protein partner or by the second subunit in case of homodimerization. Alternatively, the catalytic residue(s) can be stabilized by a protein partner (or a second subunit in the case of a dimer) when they are already present in an otherwise flexible part of the active site [3], [4].

With the genome-sequencing era, the presence of previously uncharted GTPases widely conserved in bacteria expanded our vision of this family [5]. Among them, nine proteins related to the Era/Obg subfamily were identified and six turned out to be essential for *Bacillus subtilis* growth [6], namely Bex* (Era), Obg (ObgE), YsxC (YihA), YphC (YfgK), YlqF and YqeH (these two latter proteins have no homolog in *E. coli*). Although their function was initially elusive, a wealth of biochemical data has been accumulated over the past few years showing that Era [7], [8], Obg [9], [10], [11], YsxC [12], [13], [14], YlqF [15], [16] and YqeH [17], [18] are very likely involved in ribosome assembly, although some of them might also participate in other cellular processes [19], [20]. EngA (Essential neisserial GTPase) also termed YphC or Der for ‘double Era’ [21], [22], has been also shown to participate in the ribosome biogenesis [13], [23], [24]. Furthermore, its encoding gene has been proposed to be part of the minimal bacterial genome required to sustain life [25]. Intriguingly, *E. coli* EngA has also been recently reported to interact with membrane and/or membrane proteins [26].

EngA is unique among GTPases because it bears two consecutive G domains, GD1 and GD2, followed by a *C*-terminal KH domain. Each G domain lacks the catalytic glutamine found in many GTPases (e.g. Ras) and therefore EngA belongs to a class of GTPases termed HAS-GTPases (Hydrophobic Amino acid Substituted for catalytic glutamine GTPases) [27]. Both G domains are required for interaction of EngA with the ribosomes *in vitro* [28], and for the *in vivo* function of the protein [29]. Crystal structures of EngA from *Thermotoga maritima* and *B. subtilis* with two phosphate ions bound to GD1 in the former and a GDP molecule bound to GD1 in the latter revealed two different arrangements of the G domains. Based on this, it has been suggested that a large conformational change might take place upon GTP hydrolysis by GD1, leading to a different positioning of this domain relative to GD2 and KH domains [30], [31]. This conformational change has been proposed to play a key role for the interaction of EngA with the ribosome.

Unlike most eukaryotic GTPases, which usually require additional proteins to hydrolyze and to exchange nucleotides rapidly, several bacterial GTPases involved in ribosome assembly show a relatively high intrinsic GTPase activity even in the absence of additional protein partners [32], [33]. Nevertheless, additional co-factors might be required for efficient catalysis. Indeed, Inouye and colleagues have previously reported that potassium stimulates the activity of the two bacterial GTPases MnmE and EngA from *T. Maritima*, although the mechanism underlying this activation remained unknown at that time [21], [34]. More recently, the chemical basis for this activation was unraveled for MnmE [35]. Potassium promotes a GTP-dependent dimerization of MnmE leading to a stabilization of the transition state for GTP hydrolysis. In fact, the 3-D structure of MnmE revealed that K^+^ mimics the arginine finger provided in *trans* by a GAP (e.g. for GAP/Ras complexes), and K^+^ was therefore proposed to act as a ‘chemical’ GAP [35]. A similar positioning and co-ordination mode of the K^+^ ion was recently observed for the ferrous iron transporter, FeoB, indicating the general character of this activation mechanism for these two GTPase families [36].

Here, we report the characterization of the *B. subtilis* EngA (YphC) and of its two shortened EngA variants called GD1 and GD2-KH. Our results indicate that potassium strongly stimulates the activity of both domains and congruently that of the whole enzyme. Yet, the activity of GD1, either basal or potassium-activated, is always far higher than that of GD2, and is similar to that of EngA. Accordingly, mutations targeting conserved motifs in GD1 affect more drastically the GTPase activity of EngA than similar mutations introduced into GD2. In contrast to MnmE, potassium-dependent activation is not associated to a dimerization of EngA, and thus potassium presumably helps to ideally position the catalytic residue(s) and/or the nucleotide thereby leading to a more efficient GTPase activity. We also obtained two new crystal structures of *B. subtilis* EngA with different nucleotide occupancy of each G-domain. One of them provides the first structure of a EngA GD1 in its apo form while the second structure contains a GTP analogue bound to GD2. Comparison of our structures with other structures of bacterial GTPases stimulated by potassium suggests that K^+^-activated GTP hydrolysis by GD2 is associated with a significant structural reorganization of its active site. Taken together, our data shed some light on the chemical basis of the high GTPase activity of GD1 as opposed to GD2.

## Results

Members of the EngA family are widely spread in the bacterial kingdom and a sequence alignment of EngA proteins from different bacteria is shown in Figure S1. This enzyme family is unique among GTPases as it bears two consecutive GTP-binding domains, GD1 and GD2, each containing the four conserved signature sequence motifs typically found in all GTPase proteins, G1 to G4, followed by a *C*-terminus KH domain (Fig. S1). A fifth signature, G5, is less obvious to detect from the sequence inspection but can be easily identified by structure-based alignments.

### EngA and GD1 Possess a High GTPase Activity, in Contrast to GD2

**Figure 1:**
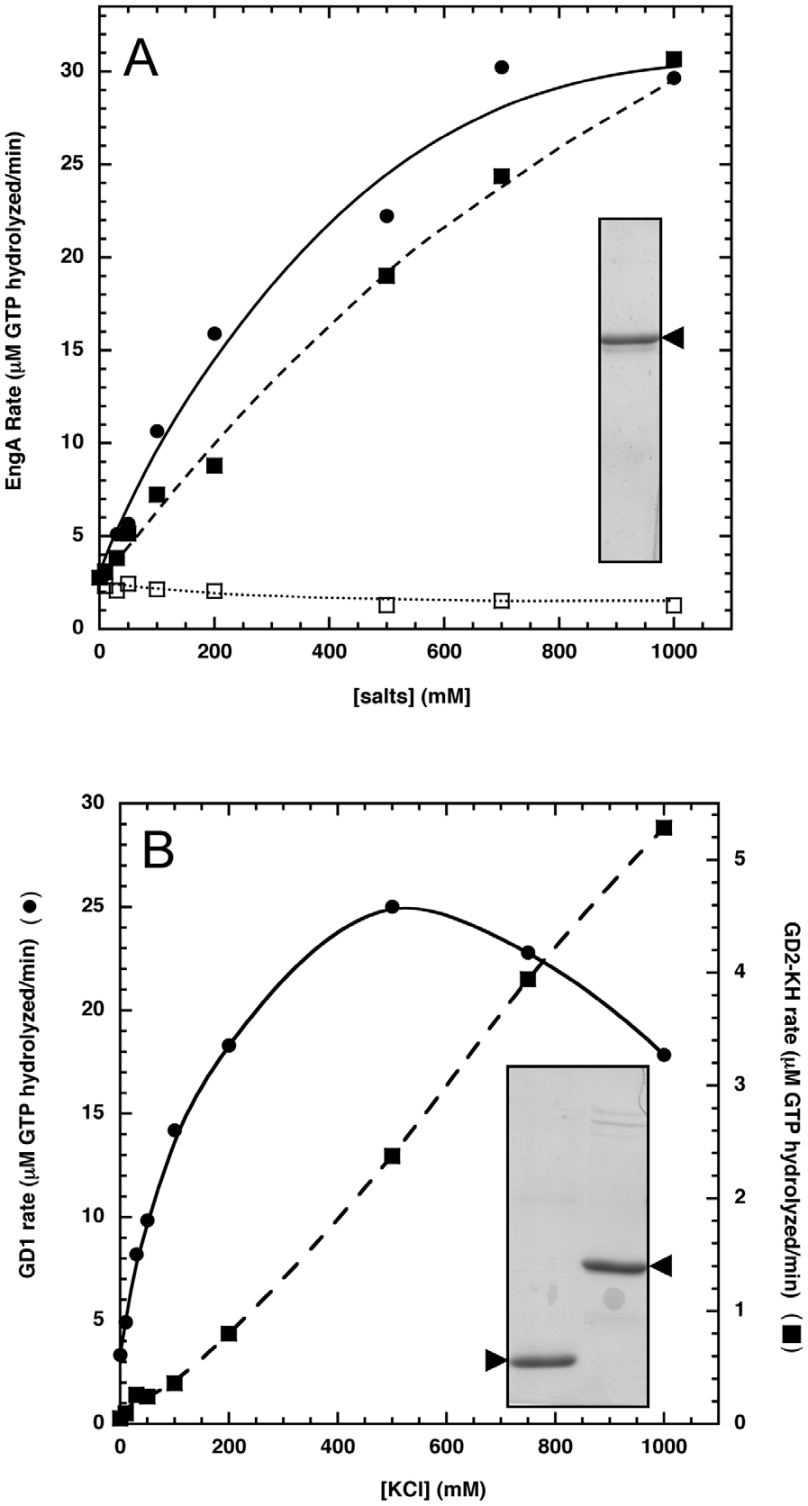
Salt effects on the GTPase activity of EngA, GD1 and GD2-KH. ***A***, the GTPase activity of EngA was monitored in the presence of 1 mM of GTP and KCl (closed circles), RbCl (closed squares), or NaCl (open squares); insert shows a SDS-PAGE of the purified EngA (indicated by a black arrowhead) which migrated close to its predicted molecular weight of 50.9 kDa). ***B***, the GTPase activity of GD1 (closed circles, left scale) or GD2-KH (closed squares, right scale) was monitored in the presence of KCl; insert shows a SDS-PAGE of the purified proteins, indicated by black arrowheads: left lane, GD1 (predicted molecular weight of 20.7 kDa); right lane, GD2-KH (predicted molecular weight of 32.1 kDa).

In order to characterize the *B. subtilis* EngA and to understand the respective role of its two GTP-binding domains, full-length EngA (named hereafter as EngA) and its two shortened EngA variants, GD1 and GD2-KH were overexpressed in *E. coli* and purified to near homogeneity (Fig. 1). A previous report on EngA from *Thermotoga maritima* had shown that its GTPase activity was strongly stimulated, about 4-fold, by K^+^ [21]. Therefore, we checked the effect of K^+^ on the GTPase activity of *B. subtilis* EngA, and test whether K^+^ was able to affect the GTPase activity of either variant. For EngA, a slow GTPase activity was measured in the absence of K^+^ (*k*_cat_ ∼0.3–0.5 min^−1^) which was strongly stimulated by addition of 300 mM potassium (Fig. S2). The GTPase activity of EngA increased to reach approximately a 15-fold stimulation in the presence of 1 M KCl (Fig. 1***A***). A similar effect was observed when K^+^ was replaced by rubidium, a slightly larger ion with similar chemical properties that often substitutes efficiently for K^+^ in biological processes [37]. In contrast, the use of the smaller sodium ion had no effect on the GTPase activity of EngA. GTPase activities of shortened EngA variants were also studied (Fig. 1***B***). In the absence of K^+^, GD1 has a GTPase activity similar to that obtained for EngA and about 150 times higher than GD2-KH (∼0.6 min^−1^ *vs* 0.004 min^−1^). Interestingly, both domains showed a strong stimulation by K^+^, with a maximal GTPase activity for GD1 in the presence of 500 mM K^+^, while that of GD2-KH increased steadily until the highest K^+^ concentration tested (1M). At 1M KCl, the GTPase activity of GD1 is ∼3.4 min^−1^ and that of GD2-KH is ∼0.45 min^−1^.

**Figure 2:**
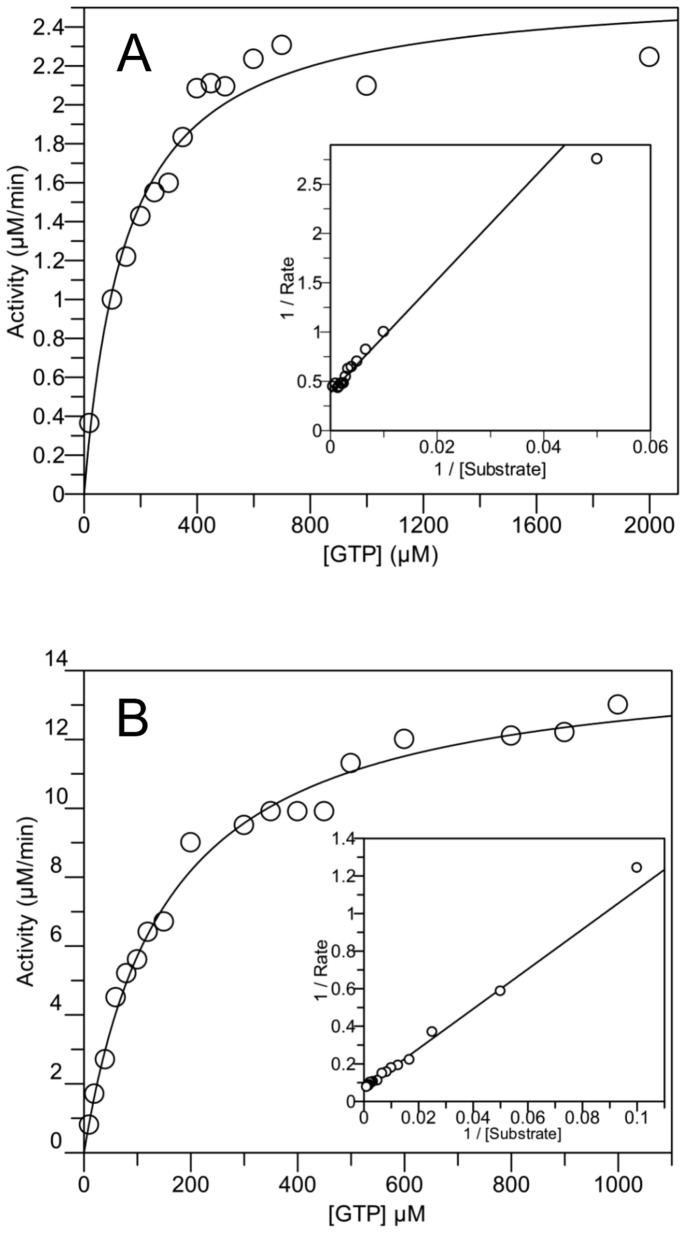
GTPase activity of EngA as a function of increasing concentrations of GTP. ***A***, in the absence of KCl; ***B***, in the presence of 300 mM of KCl. *K*_M_ and *V*_max_ values were determined from iterative nonlinear fits of the Michaelis-Menten equation to the experimental data, using the GraFit 5.0.11 software (Erithacus software) and the fitted curve are shown. The inserts show the Lineweaver-Burk plots of the data. One set of data is shown and similar results were obtained from at least three independent experiments.

The kinetic parameters of GTP hydrolysis by *B. subtilis* EngA were further investigated in the presence or absence of potassium. Rates of GTP hydrolysis were plotted against increasing concentrations of GTP and, in both cases, the best fit of the data were obtained using Henri-Michaelis-Menten equations (Fig. 2). The fitted parameters were *K_M_* = 111 µM ±14.7, *V*_max_ = 6.1±0.2 nmol/min/mg protein (*k*_cat_ ∼0.3 min^−1^) and *K_M_* = 153 µM ±11.6, *V*_max_ = 144.5±3.4 nmol/min/mg protein (*k*_cat_ ∼7.4 min^−1^) in the absence or presence of 300 mM K^+^, respectively (Table S1). Thus, K^+^ appears to impact much more on the catalytic efficiency of EngA (20-fold increase) than on its *K_M_* for GTP. The *K_M_* value found here for the *B. subtilis* EngA agrees with those reported previously for EngA of other species with values varying between 39, 110 and 143 µM for *M. smegmatis*, *T. maritima* and *E. coli* enzymes, respectively [21], [29], [38]. Yet, the *k*_cat_ values measured at 400 mM K^+^ were quite lower for *T. maritima* (0.87 min^−1^) and *E. coli* enzymes (1.16 min^−1^) [21], [29]. For *M. smegmatis* EngA and in the absence of K^+^, a *k*_cat_ of 0.005 min^−1^ was reported [38]. A comparison with other bacterial GTPases reveals that MnmE has a somewhat higher GTPase activity than *B. subtilis* EngA in the presence of K^+^ (*k*_cat_ values vary between 7.8 and 26 min^−1^) while its activity without K^+^ was similar to that reported here (*k*_cat_ = 0.33 min^−1^) [20], and FeoB in the presence of potassium has a *k*_cat_ = 0.258 min^−1^ [39]. For the other bacterial enzymes namely Era, Obg, YihA, YchF and HflX they all had a lower or even a much lower GTPase activity than *B. subtilis* EngA, but some of them might require K^+^ to be fully active [20].

### EngA, GD1 and GD2 Have Similar Affinities for Nucleotides and K^+^ Does Not Affect Them

**Figure 3:**
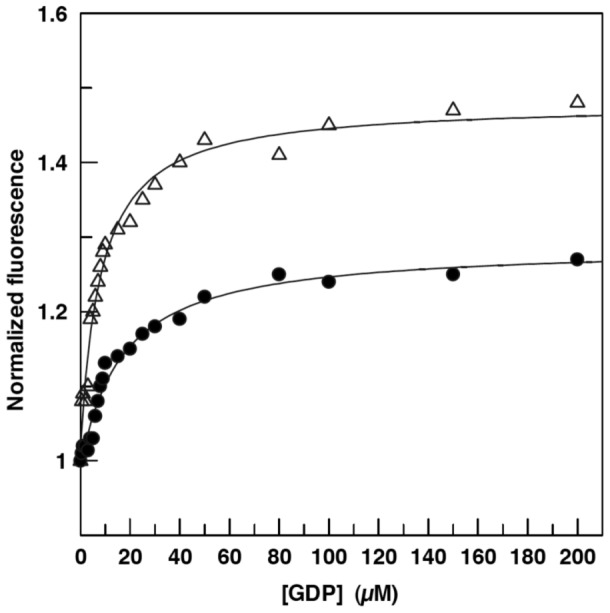
Binding of GDP to EngA. The binding of GDP was monitored by the increase in tryptophan fluorescence of EngA at 25°C, in the absence (open triangles) or in the presence (closed circles) of 300 mM KCl. The data were fitted using the GraFit 5.0.11 software and the fitted curves are shown.

To confirm the marginal effect of K^+^ on the affinity of *B. subtilis* EngA for nucleotides, fluorescence experiments were performed. *B. subtilis* EngA contains two Trp residues in its sequence (Fig. S1 and S3) and, upon excitation at 295 nm, the protein displayed a classical Trp fluorescence spectra (Fig. S4). As shown in Figure 3, addition of increasing GDP concentrations produced an increase in the intrinsic Trp fluorescence of EngA, which was more pronounced in the absence of K^+^. As observed for the kinetic experiments, a single class of binding sites was sufficient to obtain a reliable fit of the data, whether or not K^+^ was present. The two K_D_ values were in the same range: 8.0±1.0 µM in the absence of K^+^ and 16.3±2.3 µM in the presence of K^+^ (Table S2). Affinities for GMPPNP, a non-hydrolysable GTP analogue, or GDP plus AlFx, a powerful inhibitor of GTPases [40], were also measured by the same technique, and no major differences were found when K^+^ was added (Table S2). Fluorescence resonance energy transfer was also analyzed between the tryptophan residues and the MANT moiety of the MANT-GDP, and again, the presence of K^+^ did not affect the *K*_D_ of EngA for this nucleotide analogue (Table S2).

**Figure 4:**
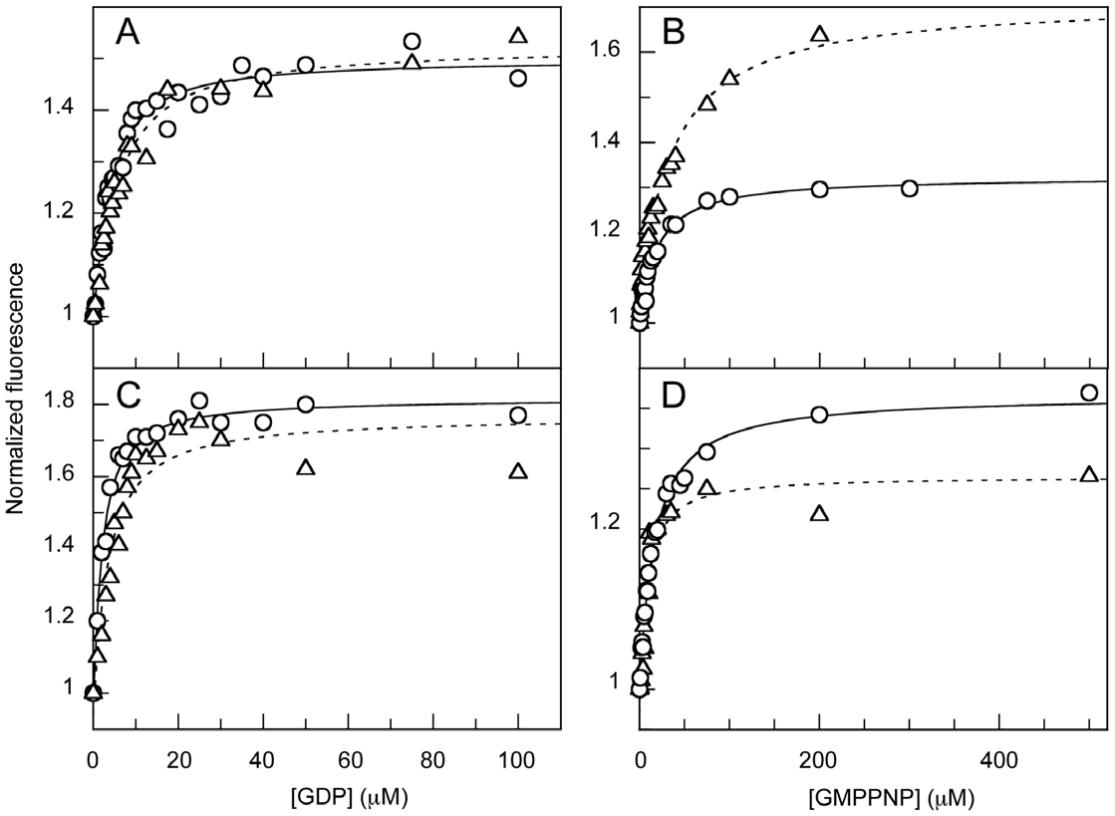
Binding of GDP and GMPPNP to GD1 and GD2-KH. The binding of GDP (***A*** and ***C***) or GMPPNP (***B*** and ***D***) was monitored by the increase in tryptophan fluorescence of GD1 (***A*** and ***B***) and GD2-KH (***C*** and ***D***) in the presence of 300 mM KCl (white triangles, dashed lines for the fitted curves) or 300 mM NaCl (white circles, plain lines for the fitted curves).

Given the presence of two potential GTP-binding sites in EngA, it was rather unexpected to detect only one class of binding sites for each nucleotide by fluorescence measurements. Possible explanations for this are (i) GD1 and GD2 have quite different affinities for one type of nucleotide and one binding site escapes the detection limits because a limited range of nucleotide concentration could only be used here (< mM), (ii) both domains have similar affinities for a given class of nucleotide, or (iii) the modification of fluorescence is due to nucleotide binding to only one of the two sites (i.e. binding of nucleotide to the second site does not produce a significant modification of Trp fluorescence). To answer this question, the same approach was applied to isolated shortened EngA variants, GD1 and GD2-KH, as they each contain a single Trp residue (Fig. S3) and they both displayed a classical Trp fluorescence spectra (Fig. S4). Upon nucleotide binding, each domain showed a marked increase in Trp fluorescence indicating that the conformation and/or environment of each Trp residue is affected by this process. Therefore, these two Trp residues are ideal reporter probes for this binding step. As shown in Figure 4, in the presence of 300 mM NaCl GD1 binds GDP and GMPPNP with *K*_D_ values of 2.7±0.4 µM and 17.8±2.4 µM, respectively (Table S2). In the presence of 300 mM KCl, these values were slightly increased to 4.5±0.9 µM and 35.7±4.9 µM for GDP and GMPPNP, respectively. For GD2-KH, similar values were obtained for the two nucleotides with *K*_D_ values of 1.6±0.2 µM (2.9±0.8 µM in the presence of K^+^) and 15.2±1.3 µM (6.8±2.0 µM in the presence of K^+^) for GDP and GMPPNP, respectively (Fig. 4 and Table S2). Therefore, the presence of K^+^ does not impact significantly on the affinity of EngA, GD1 or GD2-KH for the different nucleotides and, overall, the shortened EngA variants bind nucleotides with affinities in the same range than the whole enzyme.

**Figure 5:**
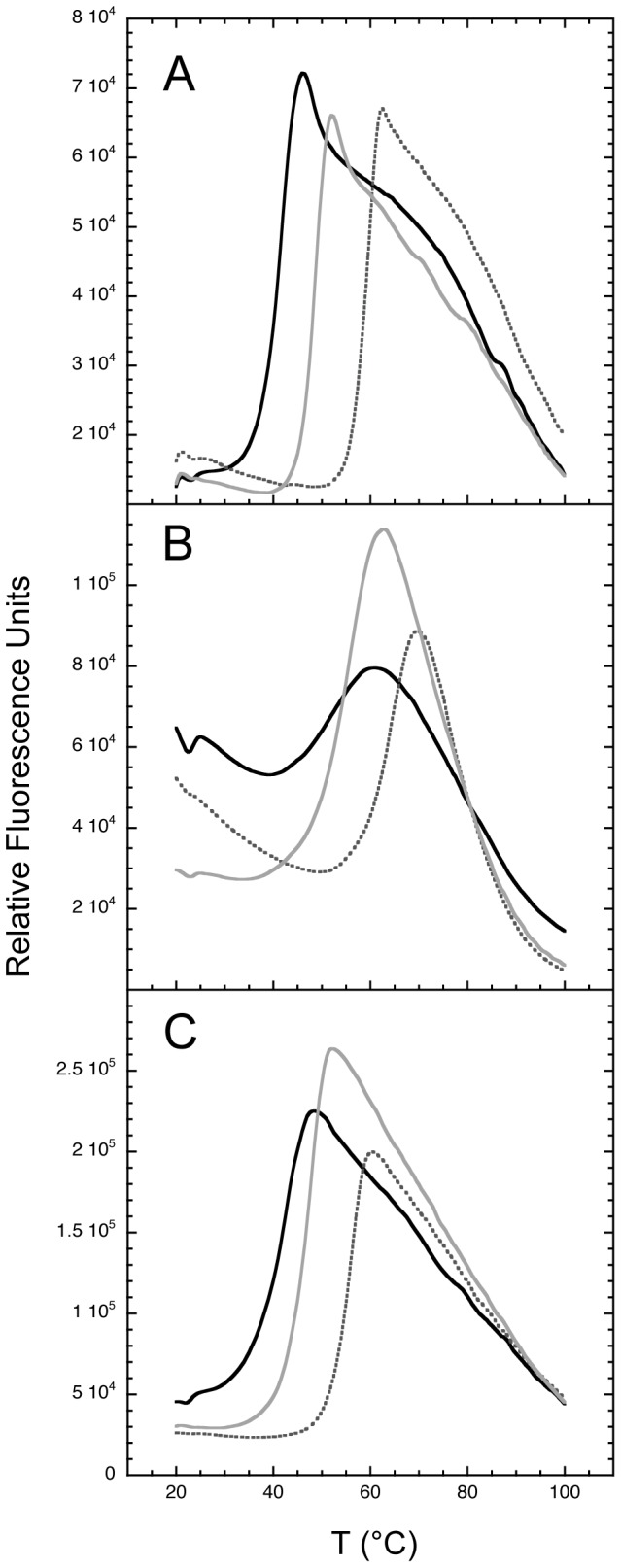
Thermal denaturation of EngA, GD1 and GD2-KH and protective effects of nucleotides. The denaturation of EngA (***A***), GD1 (***B***) and GD2-KH (***C***) was monitored by the Thermal Shift Assay in the absence (plain black lines) or in the presence of 1 mM GMPPNP (plain grey lines) or 1 mM GDP (dotted grey lines).

To further assess the binding of nucleotides to EngA and to its isolated domains, the Thermal Shift Assay (TSA) was also used. In this technique, above a given temperature threshold, the protein starts to unfold thus exposing its hydrophobic patches to the solvent. These newly exposed areas bind the fluorescent probe thereby boosting its fluorescence intensity. Consequently, the unfolding process can be monitored by the fluorescence increase as a function of temperature, with the mid-point between the folded and unfolded protein states defining the melting temperature, *Tm* [41], [42]. This technique can also be used to monitor the protein stability induced by various effectors [41], [43]. In the absence of nucleotide, EngA had a *Tm* of 41.5°C, and this value increased to 48.8°C and 59.4°C in the presence of 1 mM of GMPPNP or GDP, respectively (Fig. 5***A*** and Table S3). Likewise, apo GD1 and GD2-KH which had a *Tm* of 51°C and 42.8°C, respectively, were significantly protected against thermal denaturation by either GMPPNP (∼4.5°C *Tm* increase for both domains) or GDP (∼13°C *Tm* increase for both domains; Fig. 5***B, 5C*** and Table S3). GDP appears then to be much more efficient than GMPPNP to increase the thermal stability of each protein construct, consistent with a seemingly tighter GDP binding, as observed for other bacterial GTPases.

### K^+^ Does Not Promote the Dimerization of EngA or GD1

**Figure 6:**
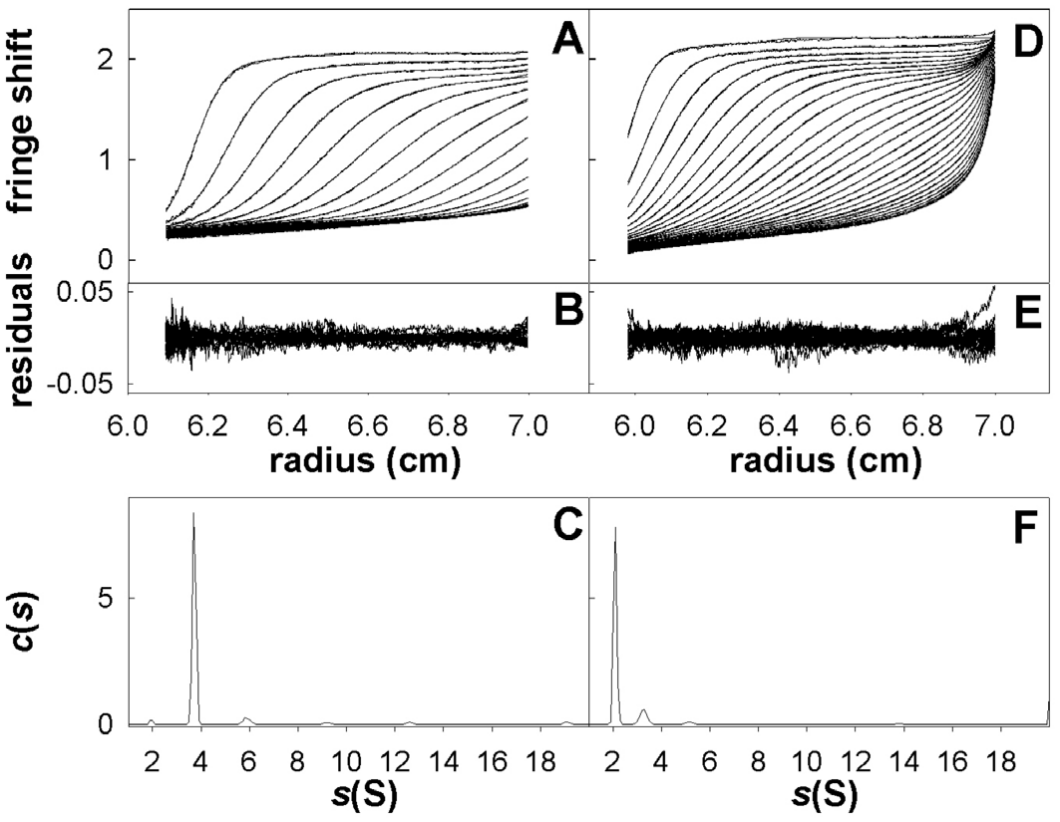
Ultracentrifugation experiments of EngA or GD1. Superposition of selected sedimentation profiles obtained at 278 nm and the corresponding fitted curves using continuous size distribution analysis for EngA (***A***) and GD1 (***D***). Superposition of the residuals (***B***, EngA; ***E***, GD1). The fitted curves were used to calculate the sedimentation coefficient distribution of the enzyme, c(s) (***C***, EngA; ***F***, GD1).

GDP-Al-F_x_ and GTP were previously shown to trigger the dimerization of the two GTPase domains of MnmE in a potassium dependant manner [35]. Therefore, we checked whether K^+^ might act in the same manner for EngA. First, gel filtrations were performed in the presence, or not, of potassium and different nucleotides: GMPPNP, GDP and GDP-Mg-AlF_x_; this latter complex is a transition state analogue [40] that was mandatory to stabilize the interaction between the two GTPase domains of MnmE in the presence of potassium [35]. Regardless of the conditions used, EngA always eluted from the gel filtration with an estimated molecular weight corresponding to a monomer (Fig. S5). Yet, transient dimers might not have withstood gel filtration so analytical ultracentrifugation experiments were also performed. Clearly, the major species obtained (∼90%) in the presence of GDP-Mg-AlF_x_ plus K^+^ for either EngA (at 3.67 S) or GD1 (at 2.09 S) corresponded to a monomer (Fig. 6). Predicted values are 3.67 S and 2.05 S, respectively, for globular monomers *versus* 5.82 S and 3.25 S for dimers. Importantly, this result was unaffected by varying the protein concentration between 1.4 and 4.7 mg/ml for EngA, and between 1.2 and 4.5 mg/ml for GD1. Similar results were also obtained in the presence of K^+^ alone, or K^+^ and GDP or GMPPNP (not shown). Therefore, in contrast to MnmE, potassium does not seem to promote the dimerization of EngA or GD1 alone.

### Equivalent Mutations Are More Drastic When They Target GD1 Rather Than GD2

**Figure 7:**
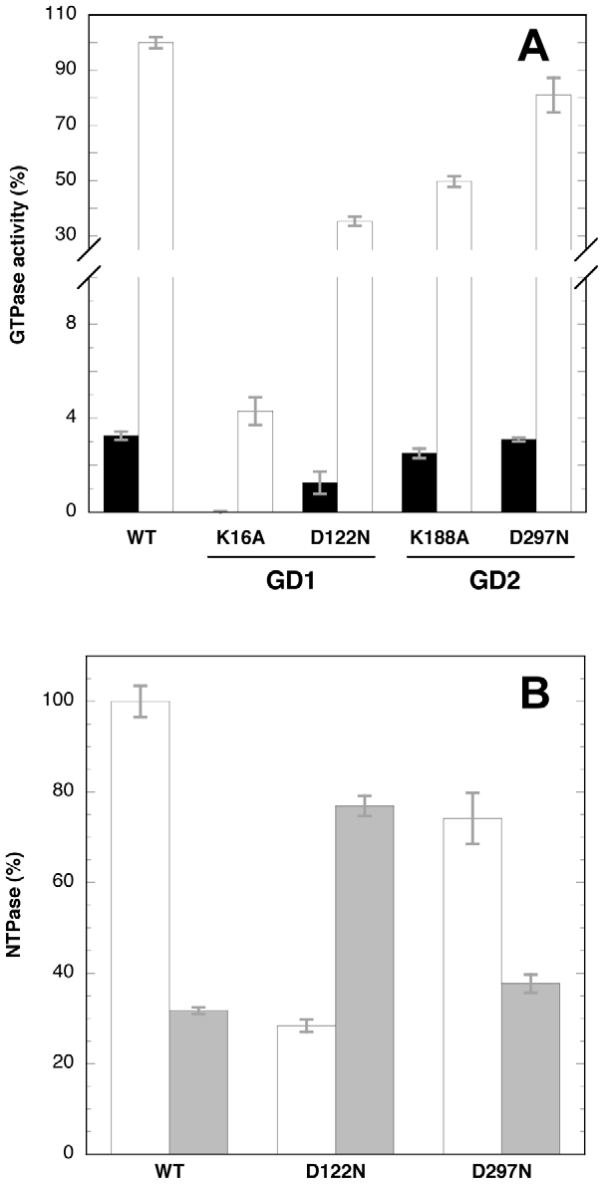
GTPase or XTPase activities of wild-type or mutated EngA. ***A***, the GTPase activity of the wild-type EngA (WT) or the mutants was measured in the presence of 1 mM GTP using a coupled-enzymatic assay in the absence (black bars) or in the presence of 300 mM KCl (white bars); the results are expressed relative to that of the wild-type EngA in the presence of 300 mM KCl (100% corresponding to 100 µmol GTP hydrolyzed/min/mg proteins). The activity of the K16A mutant is undetectable in the absence of potassium. ***B***, same as ***A*** but 200 µM of nucleotides were used in the presence of 300 mM KCl (100% corresponding to 77 µmol GTP hydrolyzed/min/mg proteins); white bars, GTPase activity; grey bars, XTPase activity. The values represent the mean of three experiments and the standard deviation is indicated as error bars, in grey color.

To further address the role of GD1 and GD2 in the context of the whole protein, identical mutations were introduced into the G1 or G4 motifs of either G domain (Fig. S1). In the G1 motifs of EngA (GX_4_GKS), the two invariant lysine residues are known to coordinate the phosphate of nucleotide and mutation of this residue to an alanine into GD1, as opposed to GD2, strongly reduced the GTPase activity of *E. coli* EngA [29]. Concerning the G4 motif (NKXD), mutation of the invariant aspartate to an asparagine was previously shown to switch the specificity in other GTPases, e.g. rasP21, EF-Tu or FtsY [44], [45], [46], thereby favoring the binding of xanthine nucleotide over guanine nucleotide. Here, mutation of the conserved Lys residue to Ala in the G1 motif of GD1 (K16A) drastically altered the GTPase activity of EngA while the equivalent mutation in GD2 (K188A) reduced the activity to ∼50% (Fig. 7***A***). The mutation Asp to Asn in the G4 motif decreased significantly the GTPase (to ∼40%) when introduced into GD1 (D122N) while it modestly affected the activity (to ∼80%) when introduced into GD2 (D298N). Because these mutations were expected to modify the nucleotide specificity, their possible gain-of-function for the XTPase activity was also studied. At 200 µM nucleotide concentration, the GTPase activity of the wild-type EngA was about three times higher than its XTPase activity (Fig. 7***B***). The same trend was observed for the D298N mutant although less pronounced than for the wild-type. Importantly, a reverse result was found with the D122N mutant with a XTPase activity more than two times higher than its GTPase activity. Overall, these results suggest that in EngA, GD1 has a much higher GTPase or XTPase activity than GD2 because (i) equivalent mutations have a more drastic effect, especially for the lysine residue of the G1 motif, when introduced into GD1 than into GD2 and (ii) a higher XTPase than GTPase activity was obtained only when the mutation switching the nucleotide specificity was introduced into GD1. However, the 50% reduced GTPase activity for the EngA K188A mutant is at odds with the activity of the GD1 domain that is estimated to be ∼40 times higher than that of the GD2 in the conditions used for the assay. Therefore, in the context of the whole enzyme, mutations in GD2 somehow also impact the activity of the GD1 domain.

### Structure of *B. subtilis* EngA With GD1 in Its Apo Form or With GMPPCP in GD2

Previously, the structure of *B. subtilis* EngA was solved with one GDP molecule in each G domain [30] (PDB code: 2HJG). Here, attempts to crystallize EngA or its isolated domains in different states were made. While we have not been able to obtain diffracting crystals for either GD1 or GD2-KH so far, suitable crystals of EngA were obtained in two different conditions. The first structure, EngA1, was obtained in the presence of ammonium sulfate and GDP and was refined to a resolution of 2.25 Å. The second structure, EngA2, was obtained without ammonium sulfate and in the presence of GMPPCP, and refined to a resolution of 2.6 Å. The two structures were obtained from crystals belonging to the same space group and with similar unit cell dimensions and crystal packing, when compared to the 2HJG entry (Table S4). Both structures had unambiguous electron density for the whole polypeptide chain, except for residues 30–40 (switch I of GD1), 60–67 (part of GD1 switch II), 122–131 (part of the GD1 G4 motif) and 206–211 (switch I of GD2). These regions were also disordered in the *B. subtilis* EngA structure determined previously [30]. The content of nucleotide-binding sites was also well defined in the electron density maps. The EngA1 structure contains one sulfate ion in GD1 and one GDP in GD2. In the EngA2 structure, the GD2 nucleotide-binding site is fully occupied by a GMPPCP molecule whereas the GD1 nucleotide-binding site remains empty. Despite the different nature of the molecule bound to the two G domains, the overall structures obtained are very similar to that previously published [30], with r.m.s differences of 0.42 Å (379 Cα atoms) and 0.31 Å (378 Cα atoms) for EngA1 and EngA2, respectively. When the GD1 in EngA1 or EngA2 are superposed to the GD1 in 2HJG, the position of the GD2 and the KH domains do not differ by more than 0.9 Å in translation and 1.3° in rotation. This clearly indicates that no major structural change occurred in its global arrangement (Fig. S3). The conformation of the GD1 in either its apo- (EngA2) or GDP-bound forms (2HJG) are nearly identical (r.m.s. difference of 0.3 Å) and the presence of a sulfate ion that takes the place of the β-phosphate (EngA1) results in a GD1 conformation also very similar to the diphosphate nucleotide bound state. (Fig. S6 ***A***). However and quite unexpectedly, the presence of a GTP analogue bound to the GD2 in EngA2 structure did not lead to any significant conformational changes (r.m.s. difference of 0.25 Å). A 1 Å shift in the switch II region (residues 236–242) is the only significant structural difference, and is likely induced by the presence of the GMPPCP γ phosphate close to it (Fig. S6B). Apparently, the binding of GMPPCP is not sufficient to trigger the expected conformational change in the GD2 switch regions. The crystallization conditions and/or the crystal packing may be partly responsible for maintaining the *B. subtilis* EngA in a GDP-bound like state. The EngA1 and EngA2 structures also suggest that the GD2 binds either GDP or GMPPCP more efficiently than the GD1, corroborating observation made for the *T. maritima* EngA structure where a GDP molecule was found in G2 while no nucleotide was added during the purification and the crystallization of the enzyme [30], [31]. However, as indicated above, the GD1 and GD2 affinity for GDP and GTP are similar in solution. Thus, this apparent inconsistency may come from different kinetics of binding or from the fact that the crystallization process may preferentially select EngA molecules that contain a nucleotide bound to GD2. Attempts to obtain an EngA structure with either a bound Mg^2+^ or a bound K^+^ ion in the presence of different nucleotides led to nearly identical structures than those reported here with no identifiable electron density for Mg^2+^ or K^+^ ions. These ions were also not reported in the two other EngA structure previously published, despite the presence of Mg^2+^ [30], [31] or K^+^ [30], [31] in the crystallization conditions.

## Discussion

Many enzymes often require co-factors for an optimal functioning and monovalent cations, especially potassium, have long been known to be required for a wide variety of biochemical pathways [47]. Early on, these monovalent cations were foreseen to form a ternary complex with the enzyme and substrate [48]. This has been recently demonstrated for one bacterial GTPase, MnmE, where K^+^ elegantly substituted for an arginine finger that is usually provided in *trans* by a GAP in many eukaryotic GTPases [35]. MnmE works as a homodimer and K^+^ in the presence of GTP (or GDP/AlFx) promotes the dimerization of the two GTP-binding domains provided by each monomer. For EngA, we found no evidence of K^+^-promoted dimerization but the idiosyncratic presence of two GTP-binding domains within a single polypeptide chain makes this enzyme more complicated to study. Thus, to elucidate the role of potassium, we have studied GD1 and GD2-KH separately. Clearly, K^+^ strongly stimulates the GTPase activity of either protein construct showing that it binds independently to both GD1 and GD2. While our study was in progress, three other families of bacterial GTPases were shown to be also stimulated by potassium: the circularly permutated GTPase (cpGTPase) and RbgA, both being involved in ribosome biogenesis [49], [50], and the G protein-coupled ferrous iron transporter B, FeoB [39]. Interestingly, K^+^ did not seem to promote the dimerization of cpGTPase [49], while the oligomerization state remains unclear for FeoB [51] and this property was not investigated for RbgA [50]. Therefore, our data show that K^+^ is a genuine EngA GTPase-activating element sufficient for activating the GTP hydrolysis of both GD1 and GD2 domains independently of a dimerization process. All K^+^-sensitive bacterial GTPases studied so far including EngA require few hundreds millimolar potassium to be fully activated and this is consistent with the intracellular concentrations found in *E. coli* (∼200 mM) or in *Bacillus subtilis* (∼400 mM) [52], [53]. Apart from cpGTPase, combined structural and phylogenetic analyses have suggested that several members of the TrmE-Era-EngA-Septin like superfamily [54] are possibly K^+^-sensitive GTPases [20], [35], [39]. Importantly, the comparison of sequences and structures of members of this superfamily identifies two conserved asparagine residues involved in the binding of potassium (see Fig. 1 in [39]). The first asparagine located within the G1 motif (GXXNXGKT/S) makes a direct contact with K^+^ in MnmE and FeoB [35], [36]. The second asparagine located 5 residues downstream of the conserved lysine residue in the G1 motif makes two hydrogen bonds with the switch I motif thereby favoring the stabilization of a K-loop binding site [36], and this K-loop was only observed in the GTP-bound conformation of FeoB [39]. Although cpGTPases have a unique topology among GTPases, YqeH does possess these two conserved asparagine residues at the expected position in its sequence (see Fig. 1 in [49]). In EngA, the switch I and therefore the putative K-loop is disordered in the crystal structures available so far, even in our structure of *B. subtilis* EngA with a GMPPCP bound to GD2. Yet, the strict conservation of these two asparagine residues in either GD1 or GD2 (see Fig. S1) combined with our observation that the GTPase activity of each G-domain of EngA is truly activated by K^+^, suggest that this activation occurs through a mechanism similar to that described for MnmE and FeoB, with each switch I of EngA adopting a K-loop conformation when GTP is bound to the two G domains.

In a previous report, the GTPase activities of GD1 and GD2 moieties of EngA from *T. maritima* have been investigated [31]. In agreement with our study, the GTPase activity of *T. maritima* GD1 approximated to that of the full-length protein. However, *T. maritima* GD2-KH retained about one third of the GTPase activity of the full-length enzyme. This contrasts with the *B. subtilis* GD2-KH that has only a marginal GTPase activity but this difference might be attributable to the intrinsically low GTPase activity of the thermostable enzyme (0.11 min^−1^ in the presence of 0.4 M KCl *vs* ∼5 min^−1^ for *B. subtilis* EngA at the same salt concentration). Remarkably, the activity of *B. subtilis* EngA without K^+^ is similar to that of *T. maritima* at 400 mM KCl. For the *B. subtilis* GD2-KH domain, the activity without K^+^ is very low and similar to that of human Ras [55]. In the presence of 300 mM K^+^, it reaches 0.11 min^−1^, a value comparable to that of full-length *T. maritima* EngA or some bacterial GTPases, e. g. Era or Obg [20]. Shortened EngA variants have been also used to decipher the multiple interactions of EngA with ribosomes [28], [38], and it has been shown for instance that the KH domain alone can bind to the 30S subunit while GD2-KH in the presence of GMPPNP binds preferentially to the 50S subunit [28]. For the whole EngA, the interactions with the ribosomal subunits seem to depend on the nature of the nucleotide bound to each G domain [28] (see below).

Since the affinity for nucleotides may impact the GTPase activity, we have characterized the binding abilities of GD1 and GD2 for different nucleotides. In a previous study, the binding affinity of *S. typhimurium* EngA for nucleotides was studied using a microcalorimetry approach [56]. While the two G-domains were found to have a similar affinity for GDP (*K*_D_ ∼1–3 µM), they exhibited a quite dissimilar affinities for GTP: one with a *K*_D_ of ∼12 µM and the other with a *K*_D_ >100 µM. Lamb and colleagues proposed that the binding site with the highest affinity reflected binding to GD1 whereas GD2 would bind GTP with a much lower affinity, and that this low affinity of GD2 for GTP might explain the small participation of GD2 in the overall GTPase activity of the enzyme [56]. Here, in the presence of Mg^2+^, we found rather similar affinities for GD1 and GD2 for either GDP or GMPPNP. Therefore, the low GTPase activity of GD2 is clearly not due to a poor binding ability of GTP in the presence of Mg^2+^. Rather, this must reflect a different catalytic proficiency of the two G-domains.

**Figure 8:**
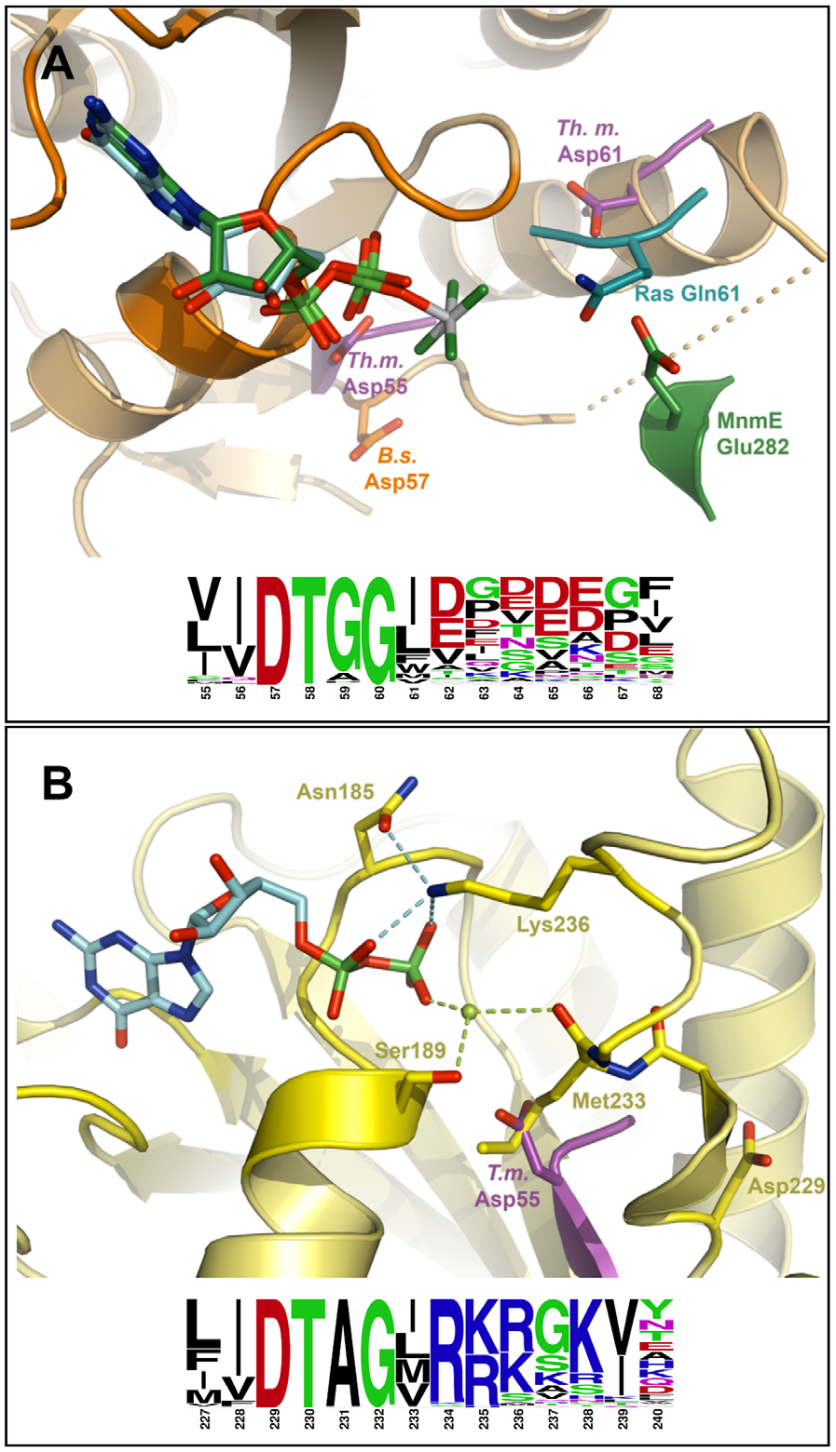
Superimposition of GD1 or GD2 domains and sequence conservation in the switch 2 motifs. ***A***, the EngA GD1 domain is depicted in orange and the GDP molecule in cyan. Gln61 from hRas is shown in turquoise, MnmE Glu282 and GDP-AlFx transition state analogue are shown in green. *T. maritima* EngA Asp55 and Asp61 are shown in purple. Structure superpositions were performed by superimposition of the G1, G4 and G5 motifs using PyMol. ***B***, the EngA GD2 domain is depicted in yellow with its bound GDP molecule shown in cyan. *T. maritima* EngA Asp55 is shown in purple. Putative Mg^2+^ position, as obtained from the superposed MnmE structure, is shown in pale green. Hydrogen bonds are depicted with cyan dashed lines. Structure superpositions were performed by superimposition of the G1, G4 and G5 motifs. One hundred EngA sequences were used to show the sequence variation in the switch 2 following the G3 motif and using the WebLogo server (http://weblogo.berkeley.edu/).

Because ultracentrifugation analysis showed that K^+^ did not promote the dimerization of GD1, residues within this domain must be self-sufficient to carry out GTP hydrolysis, and in a much more efficient way than in GD2. In MnmE, a glutamate residue in the switch II motif (Glu282, see Fig. 8***A***) is poised to stabilize a nucleophilic water molecule via a bridging water molecule, and this residue has been proposed to act as a general base to hydrolyze GTP [35]. In *B. subtilis* GD1 structures, this switch II is partially disordered but it is well resolved in GD1 of *T. maritima* where the Asp61 points towards a phosphate bound in the active site. Interestingly, although Asp61 from *T. maritima* EngA and Glu282 from MnmE are not superimposed, they are located on each side of the nucleotide and at a similar distance from the position of the γ-phosphate of GTP (Fig. 8***A***). Asp61 is not strictly conserved in GD1 from different species but acidic residues (e.g. Asp62, Asp65 and Glu66 in *B. subtilis*, all being disordered in *B. subtilis* structures) are always found in the switch II of GD1 in the EngA family (Fig. 8***A***). This is in sharp contrast with the switch II of GD2 that contains no acidic residues (Fig. 8***B***). Interestingly, in the case of FeoB no catalytic base could be identified, and instead, it has been proposed that the attacking water molecule is aligned only by the protein backbone [36]. Thus, due to the different nature of the switch II in GD1 and GD2, a different mechanism might take place in each G-domain to activate a water molecule leading to GTP hydrolysis.

In the switch II of GD2, Lys236 has been shown to contact both the α- and β-phosphate of GDP [30]. In fact, the NZ group of this lysine also occupies the same position than the arginine finger guanidinium group in the Ras-RasGAP complex structure (1 wq1 pdb entry, [57]) or the K^+^ ion in the MnmE structure [35] (Fig. S7). Assuming that K^+^ will bind to GD2 in the same way as in MnmE and FeoB, the side chain of this lysine must be reoriented to leave a free access to K^+^. Moreover, the whole switch II region of GD2 should move away in order to allow the G2 Thr210 to interact with the Mg^2+^ ion and permit the switch I to adopt the K-loop conformation. Last, as also observed in *T. maritima* EngA, the aspartic acid residue of the G3 motif (Asp229 for *B. subtilis*), is not pointing towards the nucleotide, but towards helix α8 instead, more than 10 Å away from the Mg^2+^ ion location (Fig. 8***B***). Overall, our data suggest that the GD2 domain may rely on two different GTP hydrolytic mechanisms *in vitro*: a low intrinsic activity without potassium due to an incomplete active site lacking the contribution of genuine G2 and G3 motifs, highlighted by the different available *B. subtilis* EngA GD2 structures, and a ∼100-fold increased GTPase activity upon K^+^ binding thereby leading to the proper positioning of the switch I and II regions.

Unexpectedly, we found that the binding of a GTP-like analogue (GMPPCP) to *B. subtilis* EngA GD2 led to a conformation very similar to that of the GDP-bound state. This is in sharp contrast with the major domain rearrangement found between the GTP-like conformation and GDP-bound states of GD1 [30]. At first glance, one could conclude that there is no substantial conformational change associated with the GTP bound state of the GD2 domain. However, the binding of a GTP molecule or an analogue is not always sufficient for switching the GTPase in its “on” conformation, e.g. for FeoB [58], [59]. As observed for Ras and MnmE, the presence of a GAP (Ras-GAP for ras or K^+^ for MnmE) is required for obtaining the activated conformation of the G-domain. Since our structure was obtained without K^+^, it may still retain the biological “off” conformation and presumably represents the GTP bound state in a low activity state.

Although the two GTP-binding domains of EngA are not equivalent in term of GTPase activity, they were reported to be both essential *in vivo*. Indeed, identical mutations targeting the conserved serine residue in the G1 motif and introduced in either GD1 or GD2 of *E. coli* EngA both resulted in a severe growth defect [29]. Unexpectedly, the N to D mutation introduced separately in the NKXD G4 motif of either domain, led to a different result. At low temperature (30°C), both functional GD1 and GD2 domains were required for bacterial growth while at higher temperature (42°C), either one of the two functional domains was dispensable [23]. Hence, the authors of this study concluded that with only a single functional domain, either GD1 or GD2, *E. coli* growth was maintained at high temperature. This suggests that G1 and G2 can work in an independent manner, and this is consistent with the fact that we, and others, have been unable to observe any cooperativity in GTP hydrolysis [21], [29]. Recently, a model was put forward to explain how the nature of the nucleotide bound to each G-domain might modulate the interaction with ribosomes [28] (see also [38] for a slightly modified model). In the original model, although GD1 and GD2 can work independently, they act *in vivo* in a concerted manner to bind to either the 50S subunit, the 30S subunit or the whole ribosome [28]. Congruent with this, some communication between the two G-domains is likely to occur because mutation of the conserved lysine in the G1 motif of GD2, and to a lesser extent the mutation of the G4 motif in GD2, were found here to significantly impact the overall rate of GTPase activity of the *B. subtilis* EngA. Likewise, the mutation in *E. coli* EngA of the invariant lysine or serine in the G1 motif of GD2 led to a lower GTPase activity (<50%) with a dramatically reduced *K*_M_ for GTP [29]. Thus, additional work will clearly be needed to unravel the mechanism by which these two sites talk to each other.

In conclusion, we have shown that each G domain of *B. subtilis* EngA is able to bind K^+^ and their respective GTPase activity is strongly activated by this cation. In the whole enzyme, the GTPase activity seems to primarily originate from the G1 domain although both domains need to be in a functional state (i.e. not mutated) to get a fully active EngA. Crystal structure of EngA with an apo G1 and a GDP bound to G2 has a global conformation similar to that previously published for *B. subilis* EngA with GDP in its two G domains. More surprisingly, even when a GTP analogue is bound to G2, the same overall structure is found, in contrast to the *T. maritima* EngA which presumably reflects the GTP bound state of G1. Additional structures and further biochemical characterization of this very unusual family of bacterial GTPases will be required to get a full understanding of its functioning mechanism.

Footnote: *The *B. subtilis* nomenclature is used here with the *E. coli* name given in parentheses.

## Materials and Methods

### Cloning and Site-Directed Mutagenesis

*YphC* (*engA*) was cloned into a pET15b vector and overexpressed as previously described [13]. GD1 and GD2-KH were amplified by PCR using the following oligonucleotides:

5′-GGGCATATGGGTAAACCTGTCGTAGCCATTGTC-3′ and.

5′-GCTGAGCAATGTTTTTAAAATGCTCTGCAAC-3′ primers for GD1 and.

5′-CATATGGAAGTTATTCAATTCTGTCTGATC-3′ and.

5′-GGGGCTCAGCTTATTTTCTAGCTCTTGCAAATAT-3′ for GD2-KH as 5′ and 3′ primers, respectively, and cloned into a pCR Blunt vector (Invitrogen). The gene was then sub-cloned into a pET15b (Novagen) expression vector using NdeI and BlpI restriction sites for insertion. Mutants were generated by the Quickchange protocol (Stratagene) with the pET15b-*engA* used as a template and the following oligonucleotides bearing the desired mutations and restriction sites, when indicated, for restriction screening.

K16A: 5′-CGGGAGACCAAATGTAGGAGCTAGCACAATCTTTAACCGG-3′, introducing a NheI restriction site,

K16A-rev: 5′-CCGGTTAAAGATTGTGCTAGCTCCTACATTTGGTCTCCCG-3′,

N119D: 5′-GCCTGTTGTTTTAGCGGTTGATAAGCTTGATAACACAGAAATG-3′, introducing a HindIII restriction site,

N119D-rev: 5′-CATTTCTGTGTTATCAAGCTTATCAACCGCTAAAACAACAGGC-3′,

D122N: 5′-GCCTGTTGTTTTAGCGGTTAATAAGCTTAATAACACAGAAATG-3′, introducing a HindIII restriction site,

D122N-rev: 5′-CATTTCTGTGTTATTAAGCTTATTAACCGCTAAAACAACAGGC-3′,

K188A: 5′-GACGTCCAAATGTCGGAGCTAGCTCACTTGTGAATGCG-3′, introducing a NheI restriction site.

K188A-rev: 5′-CGCATTCACAAGTGAGCTAGCTCCGACATTTGGACGTC-3′,

D297N: 5′-GTCGTAAACAAATGGAATGCTGTTGACAAAGATGAGAGC-3′, no restriction sites was either introduced or deleted,

D297N-rev: 5′-GCTCTCATCTTTGTCAACAGCATTCCATTTGTTTACGACG-3′.

The DNA template was then digested using DpnI enzyme and amplified vectors were transformed into the *E. coli* XL1 blue strain. Positive cloned were selected on LB-agar plates supplemented with ampicillin, and verified after plasmid preparation by restriction mapping and DNA sequencing (Genome express).

### Protein Overexpression and Purification

Shortened EngA variants and single point mutants were overexpressed and purified as for the wild type. C41(DE3) *E. coli* strain transformed with the vector of interest were grown until the optical density at 600 nm reached a value of 0.6–0.8, then 1 mM isopropyl-β-thiogalactopyranoside (Euromedex) was added. After overexpression for 4 hours at 37°C, cells were harvested by a low speed centrifugation (4,000 g for 10 min), resuspended in a lysis buffer (50 mM Tris-HCl pH 8, 250 mM NaCl, 10 mM imidazole, 1 mM PMSF, 5 µM leupeptine and 5 µM pepstatine A) and lysed using a French Press at 16,000 psi. The lysate was then centrifuged for 30 min at 20,000 g at 4°C to remove cell debris. The supernatant was applied onto a Ni-IDA agarose column equilibrated with the lysis buffer. The resin was washed with 10 column volumes using 50 mM Tris-HCl pH 8, 250 mM NaCl, 2.5 mM β-mercaptoethanol, 20 mM imidazole and 10% glycerol, and the protein was eluted using the same buffer except that 250 mM imidazole was used. EngA was further purified by size exclusion chromatography using a Superdex G75 16/60 (GE Healthcare) equilibrated in 20 mM Tris-HCl pH 8, 250 mM NaCl, 2.5 mM β-mercaptoethanol. Fractions containing the protein of interest were pooled and 10% glycerol was added before a flash-frozen step in liquid nitrogen. Purified proteins were stored at −80°C. Before use, the protein samples were thawed, applied onto a PD10 desalting column (GE healthcare) and concentrated using a vivaspin filter with a molecular weight cut-off of 30 kDa. The protein concentration was calculated using an extinction coefficient at 280 nm of 33350 M^−1^ cm^−1^ for EngA, 17420 for GD2-KH and 14440 for GD1, as obtained from the ProtParam tool software (http://web.expasy.org/protparam/).

### GTP Hydrolysis Assay

GTPase activities were determined using a coupled-enzymatic assay containing pyruvate kinase and lactate dehydrogenase as previously described [60]. The proteins (2 µM or 8 µM of EngA in the presence of KCl or NaCl, respectively, and 4 µM for GD1 and 12.5 µM for GD2-KH) and the coupled assay system containing 50 mM Tris/HCl pH 8, 4 mM phosphoenol pyruvate, 0.4 mM NADH, 1 mM MgCl_2_, 30 µg pyruvate kinase and 15 µg lactate dehydrogenase, were mixed into a quartz cuvette (final volume of 750 µl) in the presence of GTP (up to 1 mM) and salts, either KCl or NaCl, as indicated in the figure legends. When the GTPase and XTPase activities of the wild-type EngA and the single point mutants were studied (Fig. 7), the assay medium (final volume of 500 µl) contained 3 mM MgCl_2_ and the pyruvate kinase was increased to 120 µg to ensure an immediate conversion of XDP back into XTP. The reaction was started by adding 3.2 µM or 0.8 µM of wild-type EngA or single point mutants in the absence or presence of 300 mM K^+^, respectively. GTP or XTP hydrolysis was monitored by following the change in absorbance at 340 nm upon conversion of NADH to NAD^+^, for 10 min at 37°C, using either a Hitachi U3350 or a Safas UVmc^2^ spectrophotometer. The slope measured (ΔDO/min) was used to calculate the activity of the protein taking into account the εNADH_340nm = _6220 l.mol^−1^.cm^−1^. Control experiments were systematically performed in the absence of nucleotides (protein alone) or in the absence of proteins (nucleotides alone) and the activities measured in all cases were negligible. Between many different EngA preparations, the specific activities measured at 1 mM GTP varied between 100–130 nmoles/min/mg protein.

### Fluorescence Spectroscopy

Fluorescence measurements were performed essentially as described previously for the unmodified nucleotides or the *N*-methylanthraniloyl-GDP (MANT-GDP) analogue using a PTI QuantaMaster 4 spectrofluorimeter [61], [62]. Dissociation constants (*K*_D_) for nucleotides were calculated by monitoring intrinsic tryptophan fluorescence variation using 295 nm excitation wavelength, with a slit width of 2 nm, and scanning emission fluorescence between 310 and 400 nm (for the unmodified nucleotide) or 400 and 500 nm (for MANT-GDP), with a slit width of 4 nm. For the intrinsic tryptophan fluorescence, inner filter effect was corrected using *N*-Acetyltryptophanamide (NATA) as a reference. 1 µM NATA or 0.5 µM of YphC were mixed with 50 mM Tris/HCl pH 8, 2 mM MgCl_2_, NaCl or KCl and increasing amount of nucleotide (GDP, GMPPNP or MANT-GDP), as indicated in the figure legends. The fluorescence was normalized respective to the integrated value of the fluorescence peak obtained in the absence of unmodified nucleotide (between 310 and 400 nm) or MANT-GDP (between 400 and 500 nm). The data were fitted using the GraFit 5.0.11 software with the following equation:
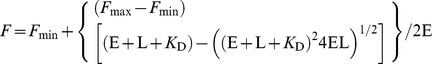
where *F* is the relative fluorescence intensity in the presence of nucleotide, *F*_min_ is the relative fluorescence in the absence of nucleotide, *F*_max_ is the fluorescence intensity at saturating concentration of nucleotide, L is the nucleotide concentration, E is the protein concentration and *K*_D_ is the dissociation constant of the protein-nucleotide complex [63].

### Fluorescence-Based Thermal Shift Assay (TSA)

Assays were performed into a 96-well thin-wall PCR plate (BioRad) using an IQ5 96-well format real-time PCR instrument (Bio-Rad) over a temperature range from 20°C to 90°C with temperature increments of 0.2°C [64]. Assay samples (23 µl) contained 50 mM Tris/HCl pH 8, 0.5 mg/ml of EngA or 1.5 mg/ml of GD1 or GD2-KH, and concentration of salt and/or nucleotides as indicated, and 2 µl of SYPRO Orange solution (Molecular Probes, Eugene, OR, 500x in DMSO diluted 5 times in water) was added. The plates were heated and changes in fluorescence for the environmentally sensitive dye SYPRO Orange were monitored through a charge-coupled device (CCD) camera [42]. The melting temperature (*Tm*) values for each protein were determined graphically from the fluorescence first derivative of the melting curves. The wavelengths for excitation and emission were 470 nm and 570 nm, respectively.

### Ultracentrifugation Analysis

Sedimentation velocity experiments were performed as previously described using a Beckman XL-I analytical ultracentrifuge equipped with an AnTi rotor [65]. Samples and buffers (400 µl and 420 µl, respectively) were loaded into their respective channels in double-sector ultracentrifuge cells and run at 45 000 r.p.m. at 4°C. Scans were recorded at 280 nm. The SEDFIT program continuous distribution c(s) analysis (Sedfit: http://www.analyticalultracentrifugation.com) [66], [67] was used to fit the data by generating the sedimentation distribution profiles. Mass identification of each peak was carried out considering EngA and GD1 as globular proteins, with Stokes radius R_h_ being 1.25 times the anhydrous radius calculated from chemical composition [68].

### Crystallization and Structure Determination

Prior to crystallization, samples of EngA were applied onto a size exclusion chromatography column (HiLoad 200 16/60, GE healthcare) equilibrated in 20 mM Tris pH 8, 250 mM NaCl, 2.5 mM β-mercaptoethanol and fractions containing pure protein were pooled and concentrated up to 8–9 mg/ml using a vivaspin concentrator with a 30 kDa cut-off. EngA samples were then mixed with either GDP or GMPPCP to reach final protein concentrations of 5 mg/ml (GDP) or 2.5 mg/ml (GMPPCP) and ligand concentrations of 10 mM (GDP) or 0.5 mM (GMPPCP). Crystals were obtained using hanging drop vapor-diffusion technique and by mixing 2 µL of EngA/nucleotide mix with 2 µL of the crystallization solution. Two different types of crystals were obtained: EngA1 (EngA/GDP mix in 4 to 10% PEG 3350, 100 mM Mes pH 6.5, 300 mM amonium sulfate), EngA2 (EngA/GMPPCP mix in 8% PEG 3350, 100 mM MES pH 6.5, 300 mM NaCl).

For X-ray diffraction data collection, crystals were first cryo-protected by soaking them in the crystallization solution to which 30% glycerol was added and then by directly frozen in liquid nitrogen. All crystallographic data were collected at −160°C on beamlines ID23-eh1 or ID23-eh2 of the European Synchrotron Radiation Facility (E.S.R.F.) in Grenoble at wavelengths of 1.282 Å or 0.873 Å using an ADSC Quantum 315R CCD detector. Data processing was performed using XDS package [69] and statistics are summarized in Table S1. The complex structures were determined by molecular replacement method with AMoRe [70] from the CCP4 suite [71] and using the EngA structure already available as search model (pdb code: 2HJG, [30]). The final models were obtained after several rounds of alternate manual building with the coot software [72] and maximum likelihood refinement with Refmac5 of the CCP4 suite [73]. The nature and the occupancy of the ligand in GD1 and GD2 domain were determined with the help of (mFobs – Fcalc) residual omit maps. EngA1 contains a sulfate ion in the GD1 nucleotide-binding site and a GDP at full occupancy in the GD2 nucleotide-binding site. For EngA2, the GD1 nucleotide-binding site is empty whereas the GD2 nucleotide-binding site is fully occupied by a GMPPCP molecule. The final refinement statistics are summarized in Table S1. The PyMol software was used to generate 3D structure figures. The structure factors and coordinates have been deposited in the Protein Data Bank (PDB) under the accession numbers 4DCS and 4DCV.

## Supporting Information

Figure S1 **Domain organization and sequence alignment of EngA.** ***A***, The boundaries of the three domains, GD1, GD2 and KH of EngA are indicated and the four motifs found in all GTPases, G1 to G4, are shown as pink and green boxes in GD1 and GD2, respectively. The mutants constructed in this study are also indicated. ***B***, alignment of some representative sequences of EngA using Clustal W (http://npsa-pbil.ibcp.fr) and ESPript (http://espript.ibcp.fr/ESPript/cgi-bin/ESPript.cgi). The abbreviations used are BACSU, *Bacillus subtilis*; THEMA, *Thermotoga maritima*; ECOLI, *Escherichia coli*; PSEAE, *Pseudomonas aeruginosa*; NEIGO, *Neisseria gonorrhoeae*; LISMO, *Listeria monocytogenes*; STARA, *Staphylococccus aureus*, MYCTU, *Mycobacterium tuberculosis*. Blue boxes are drawn when at least 50% of the residues were conserved (residues in red color with a yellow frame), and fully conserved residues are shown in white color with a red frame. The residues mutated are indicated by black arrowheads and the two Trp residues by stars. The G1 to G4 motifs are underlined in pink and green in GD1 and GD2, respectively and the secondary structure of *B. subtilis* EngA deduced from its 3D structure is shown above the sequences.
(PDF)

Figure S2 **Illustration of the GTPase activity of EngA.** The GTPase activity of EngA was monitored at 340 nm in the presence of 1 mM GTP and using a coupled-enzymatic assay to follow the change in absorbance at 340 nm upon conversion of NADH to NAD^+^, in the absence (black trace, 3.2 µM of EngA) or in the presence of 300 mM K^+^ (red trace, 0.8 µM of EngA).
(PDF)

Figure S3 **Overall view of the** ***B. subtilis*** **EngA1 and EngA2 structures superposed to the previously solved** ***B. subtilis*** **EngA structure (pdb entry 2HJG).** The EngA1 Cα backbone is depicted in pale orange (GD1), pale yellow (GD2) and pale green (KH domain). The EngA2 Cα backbone is depicted in orange (GD1), lime (GD2) and forest green (KH domain). The 2HJG Cα backbone and the GDP molecules (thin lines) bound to GD1 and GD2 are depicted in grey. The GDP molecule bound to the EngA1 GD2 is shown with cyan sticks. The GMPPCP molecule bound to the EngA2 GD2 is shown with turquoise sticks. The EngA1 sulfate ion present in the GD1 nucleotide-binding site is shown in yellow. The two tryptophan residues present in the EngA sequence have been highlighted in hot pink. The minimal GDP-tryptophan side chain distances are 13.0 Å and 8.4 Å for GD1 Trp48 and GD2 Trp296, respectively.
(PDF)

Figure S4 **Tryptophan emission spectrum of EngA, GD1 and GD2-KH.** After excitation at 295 nm, tryptophan emission spectrum of 0.5 µM EngA (***A***), 1 µM GD1 (***B***) and 1 µM GD2-KH (***C***) are shown in 50 mM Tris/HCl pH 7.5 and 300 mM KCl (red curves) or 300 mM NaCl (black curves).
(PDF)

Figure S5 **Analytical gel filtration profiles of EngA in the presence of different effectors.** Analytical gel filtrations were performed using a Superdex 75 10/30 equilibrated with a buffer containing 50 mM Tris/HCl pH 7.5 and 300 mM NaCl (***A***), 300 mM KCl (***B***) or 300 mM KCl, 1 mM GDP, 1 mM AlCl_3_ and 10 mM NaF (***C***) after a prior incubation of 0.5 mg of EngA in the same buffer during 15 min on ice. Fractions of 0.5 ml were collected and the elution volumes of albumin (65 kDa; red arrow), ovalbumin (43 kDa; blue arrow), chymotrypsin (25 kDa; green arrow) and RNase (13.7 kDa; grey arrow) are indicated. Vo corresponds to the void volume and Vt to the total volume of the column. A similar profile was also obtained when EngA was incubated in the presence of 50 mM Tris/HCl pH 7.5, 300 mM KCl and 1 mM GMPPNP and submitted to a gel filtration equilibrated in the same buffer (not shown).
(PDF)

Figure S6 **Superimposition of GD1 or GD2 nucleotide-binding site from different EngA structures.** ***A***, GD1 domains. The EngA1 is depicted in pale yellow, with the sulphate ion shown in yellow (sulfur atom). The EngA3 structure is depicted in orange with the GDP bound molecule shown in cyan. The EngA4 structure is depicted in green and *T. maritima* EngA is depicted in purple, including its two bound phosphate ions in pink (phosphorus atoms). B, G2 domains. The EngA4 GD2 is depicted in pale yellow with its bound GMPPCP molecule shown in turquoise. The EngA3 GD2 is depicted in grey, with its bound GDP molecule in cyan.
(PDF)

Figure S7 **View of the EngA GD2 switch II region and its superposition with MnmE, FeoB and Ras-RasGAP.** The EngA GD2 is shown in yellow with the switch II region highlighted in orange with its own GDP and GMPPCP, superimposed here, depicted in cyan. The FeoB K^+^ and Mg^2+^ ions are shown as semitransparent pale green and purple spheres, respectively. K-loops are shown in light blue (FeoB) and pink (MnmE) coils. Ras Gln61 and RasGAP Arg789 (arginine finger) are shown in turquoise. Hydrogen bonds stabilizing either the GD2 Lys236 NZ atom or the Mg^2+^ ion are depicted with cyan dashed lines. Structure superimpositions were performed by superimposition of the G1, G4 and G5 motifs. The EngA Met 233 carbonyl group almost superposed to the G2 Thr35 from FeoB, shown in sticks, and could contribute to stabilize the Mg^2+^ ion in the absence of a structurally stable K-loop.
(PDF)

Table S1 **Fitted Kinetic parameters of** ***B. subtilis*** **EngA obtained from** **Fig. 2** **.**
(DOC)

Table S2 **Effect of K^+^ on the affinity of EngA for different nucleotides.** ***nd*** **: not determined.**
(DOC)

Table S3 ***Tm*** **values obtained from the thermal shift experiments shown in** **Fig. 5** **.**
(DOC)

Table S4 **Crystallographic data collection and refinement statistics for EngA structures in the presence of different ligands.** ^a^Values in parentheses refer to the highest resolution shell; ^b^R_merge_ = ∑|I(h,i)-I(h)|/∑I(h,i); ^c^R = ∑|F_obs_(h,i)-F_calc_(h,i)|/∑F_obs_(h,i). R_factor_ is calculated on all reflection, R_free_ on free set reflections (10% of all reflections).
(DOC)
