# Supplementary figures and images for: Potassium Acts as a GTPase-Activating Element on Each Nucleotide-Binding Domain of the Essential Bacillus subtilis EngA
Source: PLoS One. 2012 Oct 8;7(10):e46795. doi: 10.1371/journal.pone.0046795 (PMC3466195; doi:10.1371/journal.pone.0046795)

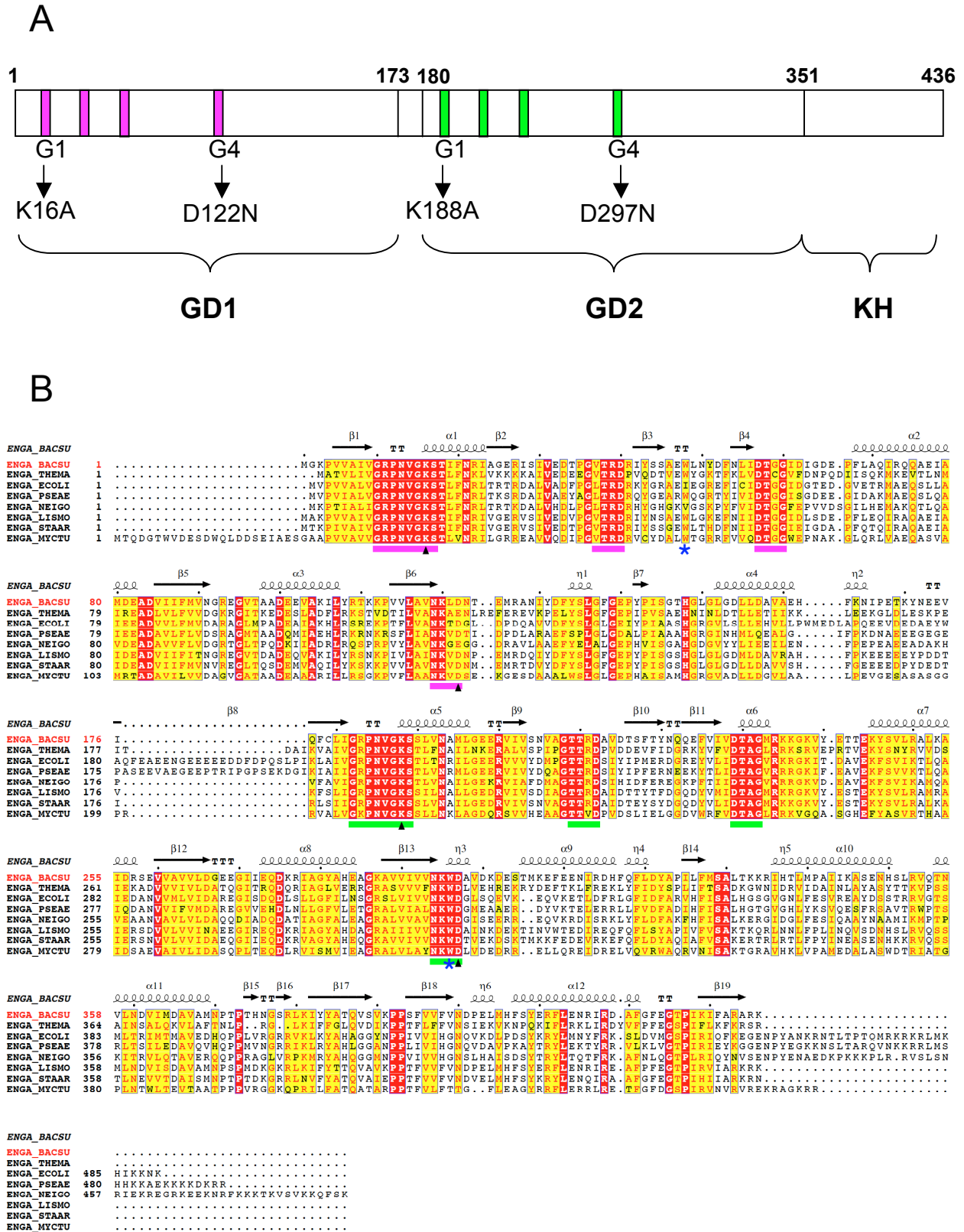

**Fig. S1**

Supplement: Figure S1 — Domain organization and sequence alignment of EngA. A, The boundaries of the three domains, GD1, GD2 and KH of EngA are indicated and the four motifs found in all GTPases, G1 to G4, are shown as pink and green boxes in GD1 and GD2, respectively. The mutants constructed in this study are also indicated. B, alignment of some representative sequences of EngA using Clustal W (http://npsa-pbil.ibcp.fr) and ESPript (http://espript.ibcp.fr/ESPript/cgi-bin/ESPript.cgi). The abbreviations used are BACSU, Bacillus subtilis; THEMA, Thermotoga maritima; ECOLI, Escherichia coli; PSEAE, Pseudomonas aeruginosa; NEIGO, Neisseria gonorrhoeae; LISMO, Listeria monocytogenes; STARA, Staphylococccus aureus, MYCTU, Mycobacterium tuberculosis. Blue boxes are drawn when at least 50% of the residues were conserved (residues in red color with a yellow frame), and fully conserved residues are shown in white color with a red frame. The residues mutated are indicated by black arrowheads and the two Trp residues by stars. The G1 to G4 motifs are underlined in pink and green in GD1 and GD2, respectively and the secondary structure of B. subtilis EngA deduced from its 3D structure is shown above the sequences. (PDF) [file pone.0046795.s001.pdf]

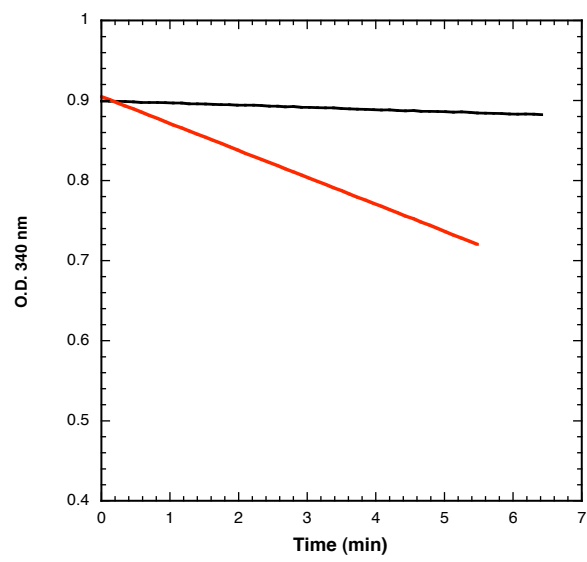

**Fig. S2**

Supplement: Figure S2 — Illustration of the GTPase activity of EngA. The GTPase activity of EngA was monitored at 340 nm in the presence of 1 mM GTP and using a coupled-enzymatic assay to follow the change in absorbance at 340 nm upon conversion of NADH to NAD+, in the absence (black trace, 3.2 µM of EngA) or in the presence of 300 mM K+ (red trace, 0.8 µM of EngA). (PDF) [file pone.0046795.s002.pdf]

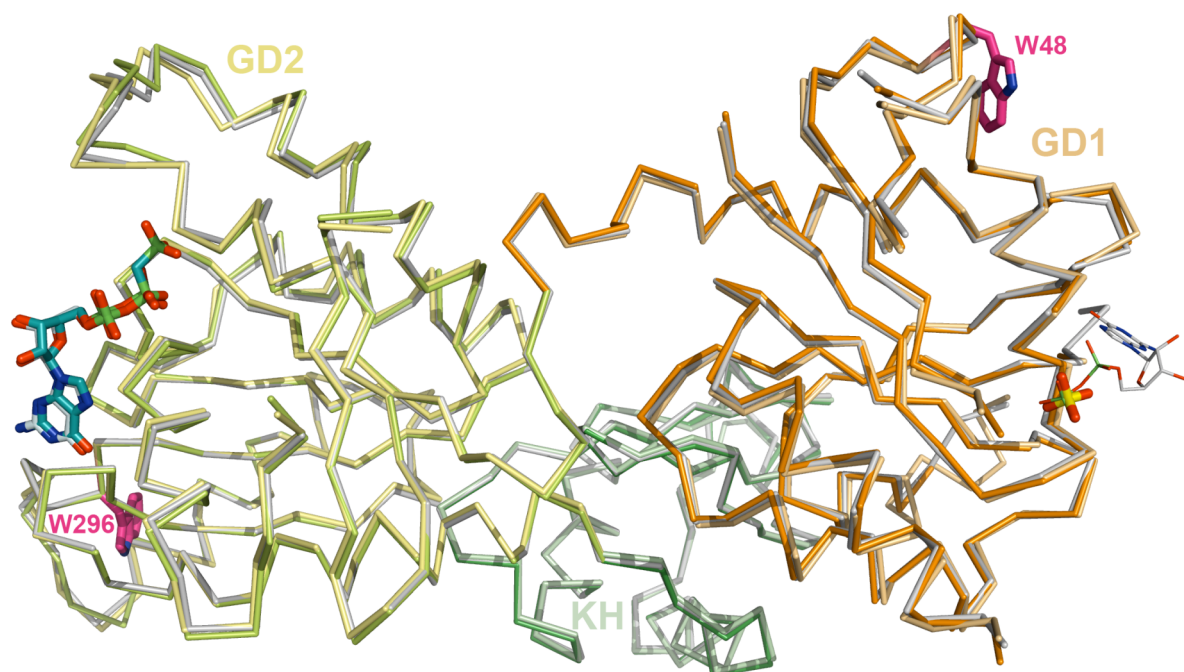

**Fig. S3**

Supplement: Figure S3 — Overall view of the B. subtilis EngA1 and EngA2 structures superposed to the previously solved B. subtilis EngA structure (pdb entry 2HJG). The EngA1 Cα backbone is depicted in pale orange (GD1), pale yellow (GD2) and pale green (KH domain). The EngA2 Cα backbone is depicted in orange (GD1), lime (GD2) and forest green (KH domain). The 2HJG Cα backbone and the GDP molecules (thin lines) bound to GD1 and GD2 are depicted in grey. The GDP molecule bound to the EngA1 GD2 is shown with cyan sticks. The GMPPCP molecule bound to the EngA2 GD2 is shown with turquoise sticks. The EngA1 sulfate ion present in the GD1 nucleotide-binding site is shown in yellow. The two tryptophan residues present in the EngA sequence have been highlighted in hot pink. The minimal GDP-tryptophan side chain distances are 13.0 Å and 8.4 Å for GD1 Trp48 and GD2 Trp296, respectively. (PDF) [file pone.0046795.s003.pdf]

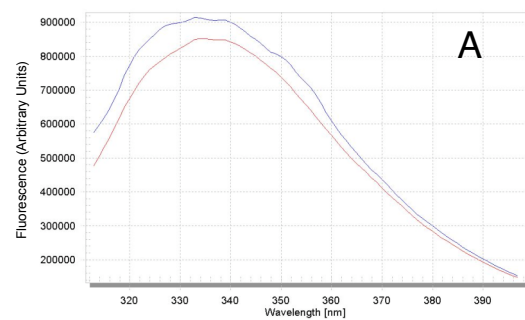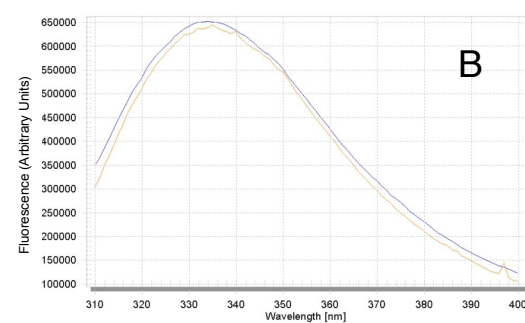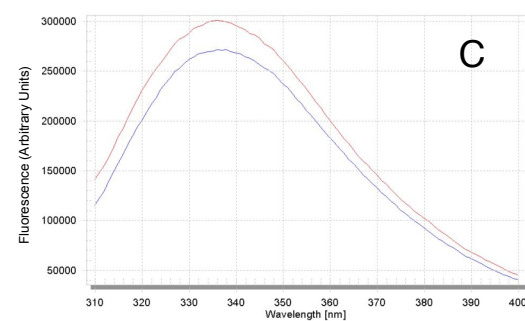

**Fig. S4**

Supplement: Figure S4 — Tryptophan emission spectrum of EngA, GD1 and GD2-KH. After excitation at 295 nm, tryptophan emission spectrum of 0.5 µM EngA (A), 1 µM GD1 (B) and 1 µM GD2-KH (C) are shown in 50 mM Tris/HCl pH 7.5 and 300 mM KCl (red curves) or 300 mM NaCl (black curves). (PDF) [file pone.0046795.s004.pdf]

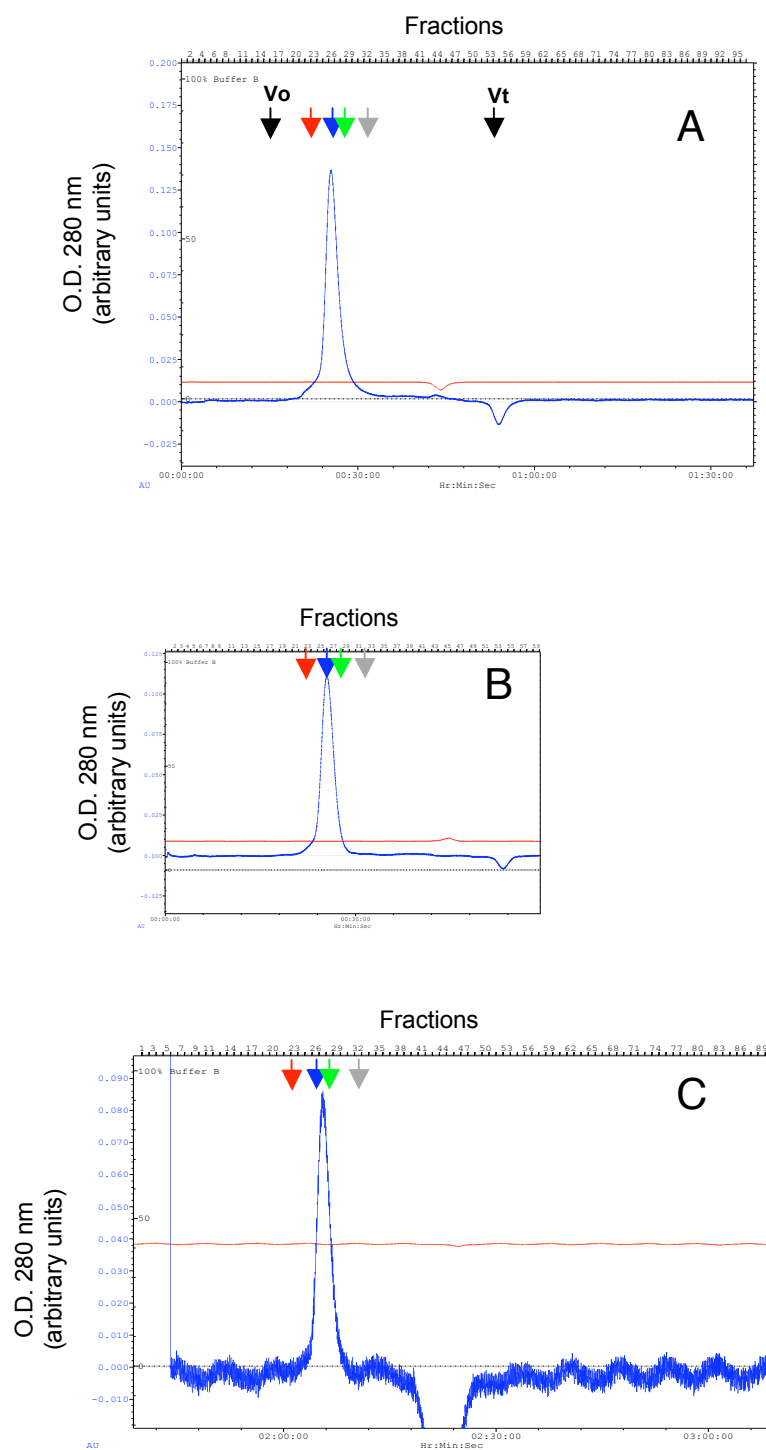

**Fig. S5**

Supplement: Figure S5 — Analytical gel filtration profiles of EngA in the presence of different effectors. Analytical gel filtrations were performed using a Superdex 75 10/30 equilibrated with a buffer containing 50 mM Tris/HCl pH 7.5 and 300 mM NaCl (A), 300 mM KCl (B) or 300 mM KCl, 1 mM GDP, 1 mM AlCl3 and 10 mM NaF (C) after a prior incubation of 0.5 mg of EngA in the same buffer during 15 min on ice. Fractions of 0.5 ml were collected and the elution volumes of albumin (65 kDa; red arrow), ovalbumin (43 kDa; blue arrow), chymotrypsin (25 kDa; green arrow) and RNase (13.7 kDa; grey arrow) are indicated. Vo corresponds to the void volume and Vt to the total volume of the column. A similar profile was also obtained when EngA was incubated in the presence of 50 mM Tris/HCl pH 7.5, 300 mM KCl and 1 mM GMPPNP and submitted to a gel filtration equilibrated in the same buffer (not shown). (PDF) [file pone.0046795.s005.pdf]

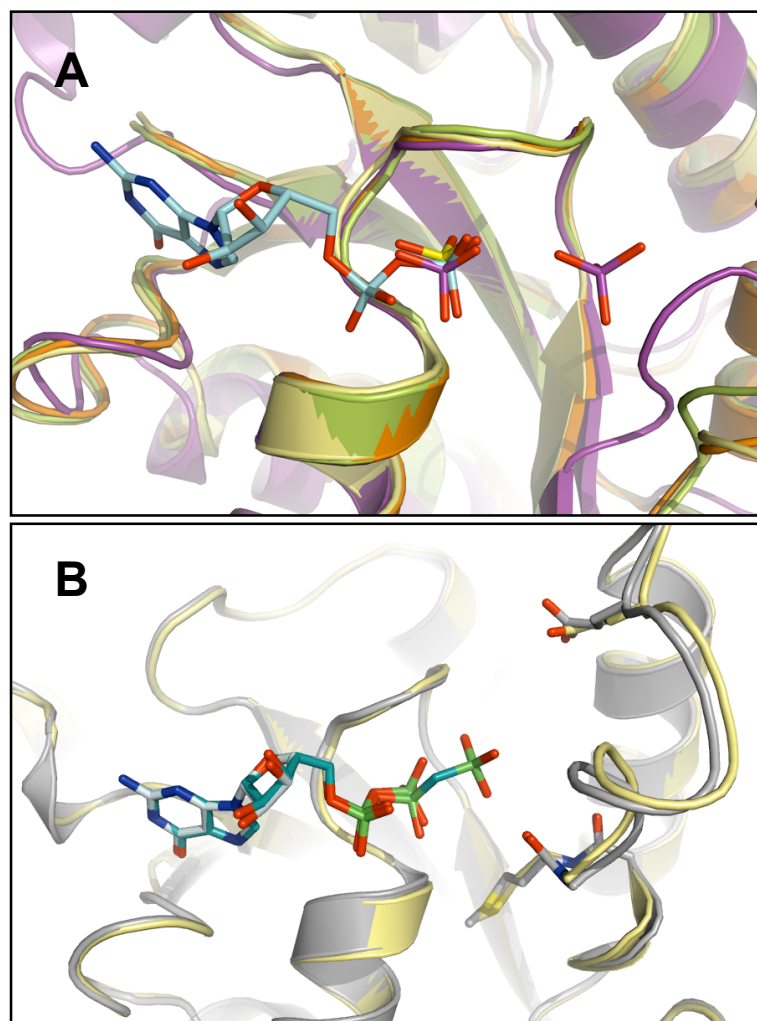

**Fig. S6**

Supplement: Figure S6 — Superimposition of GD1 or GD2 nucleotide-binding site from different EngA structures. A, GD1 domains. The EngA1 is depicted in pale yellow, with the sulphate ion shown in yellow (sulfur atom). The EngA3 structure is depicted in orange with the GDP bound molecule shown in cyan. The EngA4 structure is depicted in green and T. maritima EngA is depicted in purple, including its two bound phosphate ions in pink (phosphorus atoms). B, G2 domains. The EngA4 GD2 is depicted in pale yellow with its bound GMPPCP molecule shown in turquoise. The EngA3 GD2 is depicted in grey, with its bound GDP molecule in cyan. (PDF) [file pone.0046795.s006.pdf]

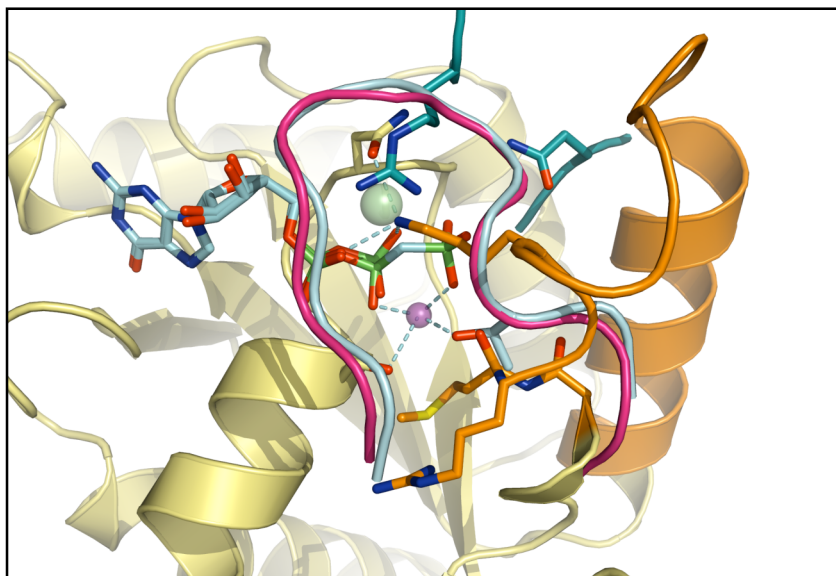

**Fig. S7**

Supplement: Figure S7 — View of the EngA GD2 switch II region and its superposition with MnmE, FeoB and Ras-RasGAP. The EngA GD2 is shown in yellow with the switch II region highlighted in orange with its own GDP and GMPPCP, superimposed here, depicted in cyan. The FeoB K+ and Mg2+ ions are shown as semitransparent pale green and purple spheres, respectively. K-loops are shown in light blue (FeoB) and pink (MnmE) coils. Ras Gln61 and RasGAP Arg789 (arginine finger) are shown in turquoise. Hydrogen bonds stabilizing either the GD2 Lys236 NZ atom or the Mg2+ ion are depicted with cyan dashed lines. Structure superimpositions were performed by superimposition of the G1, G4 and G5 motifs. The EngA Met 233 carbonyl group almost superposed to the G2 Thr35 from FeoB, shown in sticks, and could contribute to stabilize the Mg2+ ion in the absence of a structurally stable K-loop. (PDF) [file pone.0046795.s007.pdf]
