# Supplementary material for: Potassium Acts as a GTPase-Activating Element on Each Nucleotide-Binding Domain of the Essential Bacillus subtilis EngA
Source: PLoS One. 2012 Oct 8;7(10):e46795. doi: 10.1371/journal.pone.0046795 (PMC3466195; doi:10.1371/journal.pone.0046795)
Supplement: Table S1 — Fitted Kinetic parameters of B. subtilis EngA obtained from Fig. 2 . (DOC) [file pone.0046795.s008.doc]

|  | *K*_M_ (μM) | *k*_cat_ (min^-1^) |
| --- | --- | --- |
| No K^+^ | 111 ± 14.7 | 0.3 |
| 300 mM K^+^ | 153 ± 11.6 | 7.4 |

**Table S1**.
