# Supplementary material for: Potassium Acts as a GTPase-Activating Element on Each Nucleotide-Binding Domain of the Essential Bacillus subtilis EngA
Source: PLoS One. 2012 Oct 8;7(10):e46795. doi: 10.1371/journal.pone.0046795 (PMC3466195; doi:10.1371/journal.pone.0046795)
Supplement: Table S2 — Effect of K+ on the affinity of EngA for different nucleotides. nd : not determined. (DOC) [file pone.0046795.s009.doc]

|  |  | ***K*_D_ (μM)** | | | |
| --- | --- | --- | --- | --- | --- |
| **Protein** | **Salt** | **GDP** | **GMPPNP** | **GDP-AlFx** | **mant-GDP** |
| **EngA** | no salt | 8.0 ± 1.0 | 25.7 ± 2.8 | 9.3 ± 2.0 | 3.1 ± 0.2 |
|  | 300 mM KCl | 16.3 ± 2.3 | 18.8 ± 2.8 | 13.8 ± 2.3 | 2.9 ± 0.2 |
|  | 300 mM NaCl | 12.8 ± 2.9 | 16.5 ± 3.7 | *nd* | *nd* |
| **GD1** | 300 mM KCl | 4.5 ± 0.9 | 35.7 ± 4.9 | *nd* | *nd* |
|  | 300 mM NaCl | 2.7 ± 0.4 | 17.8 ± 2.4 | *nd* | *nd* |
| **GD2-KH** | 300 mM KCl | 2.9 ± 0.8 | 6.8 ± 2 | *nd* | *nd* |
|  | 300 mM NaCl | 1.6 ± 0.2 | 15.2 ± 1.3 | *nd* | *nd* |

**Table S2**.
