# Supplementary material for: Potassium Acts as a GTPase-Activating Element on Each Nucleotide-Binding Domain of the Essential Bacillus subtilis EngA
Source: PLoS One. 2012 Oct 8;7(10):e46795. doi: 10.1371/journal.pone.0046795 (PMC3466195; doi:10.1371/journal.pone.0046795)
Supplement: Table S3 — Tm values obtained from the thermal shift experiments shown in Fig. 5 . (DOC) [file pone.0046795.s010.doc]

|  | *Tm* (°C) | | |
| --- | --- | --- | --- |
|  | EngA | GD1 | GD2-KH |
| No effector | 41.5 | 51 | 42.8 |
| 1 mM GMPPNP | 48.8 | 55.5 | 48.3 |
| 1 mM GDP | 59.4 | 64 | 55.8 |

**Table S3**.
