# Supplementary material for: Potassium Acts as a GTPase-Activating Element on Each Nucleotide-Binding Domain of the Essential Bacillus subtilis EngA
Source: PLoS One. 2012 Oct 8;7(10):e46795. doi: 10.1371/journal.pone.0046795 (PMC3466195; doi:10.1371/journal.pone.0046795)
Supplement: Table S4 — Crystallographic data collection and refinement statistics for EngA structures in the presence of different ligands. aValues in parentheses refer to the highest resolution shell; bRmerge = ∑|I(h,i)-I(h)|/∑I(h,i); cR = ∑|Fobs(h,i)-Fcalc(h,i)|/∑Fobs(h,i). Rfactor is calculated on all reflection, Rfree on free set reflections (10% of all reflections). (DOC) [file pone.0046795.s011.doc]

|  |  | **EngA1** | **EngA2** |
| --- | --- | --- | --- |
| **PDB entry code** |  | **4DCS** | **4DCV** |
| ***Data collection statistics*** |  |  |  |
| **Space group** |  | ***P***2_1_2_1_2_1_ | ***P***2_1_2_1_2_1_ |
| **Cell dimension**  a= | a (Å) | 63.30 | 62.49 |
|  | b (Å) | 65.79 | 66.19 |
|  | c (Å) | 111.43 | 111.74 |
| **Resolution range (Å)^a^** |  | 20-2.25 (2.35-2.25) | 57-2.60 (2.69-2.60) |
| **R_merge_ (%)^a,b^** |  | 10.2 (40.6) | 11.0 (51.9) |
| **Completeness (%)^a^** |  | 97.3 (92.1) | 95.1 (96.3) |
| **I/σ ^a^** |  | 13.0 (5.0) | 11.4 (4.1) |
| **No. Reflexions ^a^** |  | 152528 (17514) | 58831 (5451) |
| **No. Unique ^a^** |  | 22133 (2523) | 14095 (1392) |
| ***Refinement Statistics*** |  |  |  |
| **Protein atoms** |  | 3165 | 3182 |
| **Other (GDP/SO4)** |  | 33 | 32 |
| **Water** |  | 96 | 53 |
| **nucleotide binding content (GD1/GD2)** |  | SO_4_/GDP | empty/GMPPCP |
| **Resolution range (Å)** |  | 20-2.25 | 15-2.60 |
| **R_factor_ (%)^c^** |  | 21.92 | 18.97 |
| **R_work_ (%)^c^** |  | 21.38 | 18.09 |
| **R_free_ (%)^c^** |  | 26.69 | 26.78 |
| **Rms deviations from ideality** |  |  |  |
| Bond lengths (Å) |  | 0.007 | 0.009 |
| Bond angles (°) |  | 1.147 | 1.453 |

**Table S4**.
